# Transcutaneous auricular vagus nerve stimulation in perioperative neurocognitive disorders: clinical evidence and mechanistic perspectives

**DOI:** 10.3389/fneur.2026.1963894

**Published:** 2026-10-29

**Authors:** Ruyi Xia, Yuhan Meng, Meiru Jiang, Jiahai Ma

**Affiliations:** 1 School of Anesthesiology, Shandong Second Medical University, Weifang, Shandong, China; 2 Second Clinical Medical College, Shandong Medical and Pharmaceutical University, Yantai, Shandong, China; 3 Department of Anesthesiology, Yantai Yuhuangding Hospital, Yantai, Shandong, China

**Keywords:** autonomic regulation, blood–brain barrier, cholinergic anti-inflammatory pathway, neuroimmunology, perioperative neurocognitive disorders, postoperative delirium, precision brain protection, transcutaneous auricular vagus nerve stimulation

## Abstract

**Background:**

Perioperative neurocognitive disorders (PND) are common neurological complications in older surgical patients and are associated with impaired recovery, loss of independence, and worse quality of life. Transcutaneous auricular vagus nerve stimulation (taVNS) is a non-invasive neuromodulatory intervention with plausible effects on inflammatory, autonomic, sleep, pain, and cognitive-network pathways.

**Methods:**

We conducted a PROSPERO-registered systematic review of direct perioperative randomized clinical evidence and, when outcomes were sufficiently comparable, an exploratory meta-analysis. PubMed/MEDLINE, Embase, Web of Science Core Collection, and CENTRAL were searched from inception through 5 August 2026. Risk of bias was assessed with RoB 2 and certainty with GRADE. Mechanistic and translational evidence was synthesized separately using a structured narrative approach.

**Results:**

Four randomized trials enrolled or randomized 480 participants, of whom 469 contributed to reported analyses. Two trials involving 220 participants assessed related but non-identical early postoperative cognitive-decline constructs: one used dNCR and the other investigator-defined traditional POCD. Both studies individually favored taVNS, and the individual study estimates were treated as the primary evidence. A *post hoc* exploratory cross-construct synthesis yielded an RR of 0.34 (95% CI 0.18–0.64). Because the contributing trials used non-equivalent cognitive definitions, stimulation regimens, and comparator conditions, this secondary pooled estimate should not be interpreted as representing either a single standardized cognitive outcome or the effect of a single standardized taVNS intervention. A separate multicenter trial reported postoperative delirium in 12.0% versus 25.3% of participants (risk ratio 0.47, 95% confidence interval 0.23–0.98), and another trial showed more favorable postoperative Montreal Cognitive Assessment trajectories. Beyond clinical outcomes, the broader mechanistic literature supports a multilevel neurophysiological rationale for taVNS, consistent with possible modulation of auricular vagal afferent–nucleus tractus solitarius signaling, locus coeruleus and autonomic regulation, inflammatory reflex pathways, splenic sympathetic–cholinergic immune modulation, blood–brain barrier/neurovascular unit protection, glial responses, sleep–arousal regulation, and cognitive-network plasticity. However, most mechanistic evidence remains derived from preclinical or non-perioperative studies, and no causal pathway linking target engagement to cognitive improvement has been established.

**Conclusion:**

Current evidence suggests a promising but preliminary clinical signal for taVNS in perioperative neurocognitive disorders. However, certainty remains low to very low, and routine clinical implementation is not yet supported. Available adverse-event data are insufficient to establish perioperative safety or to exclude uncommon cardiovascular or autonomic adverse events. Standardized PND outcomes, credible sham conditions, optimized stimulation dose and timing, systematic adverse-event ascertainment, multimodal target-engagement biomarkers, and risk-enriched confirmatory trials are priorities for future development.

**Systematic review registration:**

https://www.crd.york.ac.uk/PROSPERO/view/CRD420261468039, Identifier: CRD420261468039.

## 1 Background

### 1.1 Clinical burden and unmet needs in perioperative neurocognitive disorders

PND is an umbrella framework for cognitive change across distinct perioperative time windows. It includes postoperative delirium (POD), delayed neurocognitive recovery (dNCR) within 30 days after surgery, and postoperative neurocognitive disorder (postoperative NCD) from 30 days to 12 months; the historical term POCD is retained only when reproducing the terminology of an original study (1–3). POD, dNCR, postoperative NCD, and an isolated change in a screening score are not interchangeable outcomes. PND can prolong hospitalization, increase care needs, impair independent living, and contribute to persistent cognitive and functional decline (4–7).

The burden is concentrated in older and frail patients, in people with reduced cognitive reserve or pre-existing impairment, and after major or physiologically stressful operations. Current prevention strategies remain incomplete because PND reflects interacting inflammatory, vascular, autonomic, sleep, pain, and network vulnerabilities rather than a single modifiable pathway (7–10).

### 1.2 Neuroimmune dysregulation as a pathophysiological basis

Surgical injury can release DAMPs such as HMGB1 and increase circulating IL-1β, IL-6, and TNF-α. Peripheral inflammatory signals may affect the brain through neural, humoral, and vascular routes, while BBB/NVU dysfunction, microglial and astrocytic activation, oxidative stress, synaptic injury, and impaired network connectivity may converge on attention, executive function, and memory (8–26).

Perioperative sympathetic activation, reduced vagal regulation, HPA-axis activation, sleep disruption, pain, and opioid exposure may amplify this vulnerability (7, 27–33). These interacting domains provide a theoretical rationale for a neuromodulatory intervention that could influence several levels of perioperative physiology, but they do not establish that any single pathway mediates clinical benefit.

### 1.3 Rationale for perioperative taVNS

taVNS is a non-invasive intervention intended to recruit auricular vagal afferents without surgical implantation. It is repeatable and potentially deployable before, during, or after surgery, making it attractive for perioperative research (34–37). Anatomical, physiological, preclinical, and non-perioperative human studies provide biological plausibility for effects on autonomic regulation, inflammatory reflexes, sleep, pain, arousal, and cognitive networks. The direct clinical question, however, is whether a reproducible stimulation dose can engage a relevant pathway in surgical patients and produce a clinically meaningful cognitive benefit.

### 1.4 Objectives of the review

This article integrates two complementary evidence streams. First, direct perioperative clinical-efficacy evidence was synthesized using systematic-review methods, outcome-specific RoB 2 and GRADE appraisal, and an exploratory meta-analysis when event definitions and assessment windows were sufficiently comparable. Second, the broader anatomical, neuroimmune, autonomic, sleep, pain, cognitive-network, preclinical, and translational literature was synthesized narratively. The objectives were to quantify the available direct clinical signal, clarify its evidentiary limitations, examine the plausibility and limits of candidate mechanisms, and define priorities for reproducible perioperative taVNS trials.

## 2 Methods

### 2.1 Overall review design, reporting framework, and registration

The direct perioperative clinical-efficacy component was conducted as a systematic review with an exploratory meta-analysis and reported in accordance with PRISMA 2020 (38). The review was registered in PROSPERO (CRD420261468039). The anatomical, neuroimmune, autonomic, sleep, and translational literature was interpreted through a structured narrative synthesis informed by the quality domains of SANRA (39) and was not treated as if it had undergone the same PICOS, RoB 2, or GRADE process. The two evidence levels were kept separate in the Methods, Results, and Conclusions.

The PROSPERO registration prespecified separate analyses of postoperative delirium, consensus-defined dNCR, investigator-defined historical POCD, isolated cognitive-scale outcomes, and longer-term postoperative NCD, with quantitative pooling restricted to studies considered sufficiently comparable in outcome construct and assessment window. After completion of study selection, only one dNCR trial and one investigator-defined traditional POCD trial provided binary early postoperative cognitive-event data. We therefore added a *post hoc* exploratory cross-construct synthesis of these two clinically related but non-equivalent outcomes. This synthesis was not prespecified in the PROSPERO registration and is reported explicitly as a protocol deviation; no changes were made to the eligibility criteria, search strategy, or study-selection process to accommodate this analysis.

### 2.2 Information sources and systematic search strategy

PubMed/MEDLINE, Embase, Web of Science Core Collection, and the Cochrane Central Register of Controlled Trials (CENTRAL) were searched from database inception through 5 August 2026. ClinicalTrials.gov, the Chinese Clinical Trial Registry (ChiCTR), and the International Traditional Medicine Clinical Trial Registry (ITMCTR) were checked separately through the same date for protocols, ongoing studies, and completed trials without published efficacy results. Reference lists and forward citations of eligible reports and relevant reviews were examined, without language restriction. The search combined taVNS synonyms with perioperative and cognitive-outcome concepts including surgery, delirium, dNCR, postoperative cognitive dysfunction, MoCA, and MMSE. Database-specific strategies, registry cross-checking procedures, and recorded yields are provided in Supplementary Methods S1. The search strategy was not independently peer-reviewed by an information specialist. No eligible non-English full-text reports requiring translation were identified during study selection.

### 2.3 Eligibility criteria for direct clinical-efficacy evidence

Eligible studies enrolled adults undergoing surgery and compared perioperative taVNS with sham stimulation, non-vagal auricular stimulation, no stimulation, usual care, or another comparator from which the taVNS effect could be isolated. Eligible designs were randomized controlled trials reporting POD, dNCR, postoperative NCD, investigator-defined POCD, validated postoperative cognitive testing, or serial postoperative cognitive scores. Protocol-only records and registrations were retained to describe the emerging research landscape but were not included in efficacy estimates. Studies of implanted or cervical VNS, non-vagal electrical stimulation without a stated auricular-vagal target, non-comparative designs, and studies without cognitive or delirium outcomes were excluded from the direct-efficacy synthesis.

### 2.4 Study selection

Two reviewers independently screened titles and abstracts and then assessed potentially eligible full texts. Disagreements were resolved through discussion and consensus. Companion reports were linked at the study level to avoid double counting, and the most complete report was prioritized for extraction.

Companion publications, protocols, and trial-registration records were matched at the study level using registration numbers where available, together with concordance in author group, study population, recruitment setting, sample size, intervention and comparator characteristics, and study dates. When information differed between a registration record and the published report, both sources were retained for verification. Prespecified design and outcome information was taken from the prospectively available registration or protocol, whereas observed participant characteristics, intervention delivery, and outcome data were taken from the final published report. Any discrepancies relevant to outcome specification, registration timing, or selective reporting were considered in the risk-of-bias assessment rather than being silently reconciled.

### 2.5 Data extraction

Two reviewers independently extracted study design, population, surgical and anesthetic characteristics, sample size, analyzed population, stimulation site and laterality, frequency, pulse width, current intensity, duty cycle, treatment timing and duration, comparator, cognitive definitions, follow-up, event counts, continuous cognitive outcomes, biomarkers, adverse events, adherence, and registration information. Data were checked against full-text reports and trial records; disagreements were resolved by consensus. During revision, the corresponding authors of two included studies with key missing information were contacted by email to request additional methodological or numerical details, including unavailable variance information and incompletely reported stimulation or comparator characteristics. No response had been received by the revision cutoff date. No automation tool was used as a reviewer. During manuscript preparation, no generative AI tool was used to generate, rewrite, or edit manuscript text, conduct study selection or data extraction, perform risk-of-bias or certainty assessments, undertake statistical analyses, or make scientific interpretations. ChatGPT (OpenAI) was used only as a terminology-reference aid to check the meaning or conventional English usage of selected professional terms. No AI-generated text was incorporated into the manuscript. All scientific content and final wording were produced and verified by the authors.

### 2.6 Outcomes and effect measures

POD, consensus-defined dNCR, investigator-defined traditional POCD, postoperative NCD, and serial cognitive scores were treated as distinct clinical outcome constructs. The Zhou and Li trials nevertheless both reported binary measures of postoperative cognitive deterioration during the first postoperative week and were similar in their broad clinical question, namely whether perioperative taVNS was associated with fewer early postoperative cognitive deficits. Because the diagnostic frameworks used by the two investigators were not equivalent, these outcomes were not regarded as a single standardized endpoint. They were combined only in a *post hoc* exploratory cross-construct synthesis to examine whether the direction and approximate magnitude of the treatment effect were concordant across two clinically related early cognitive-decline constructs. The pooled estimate was not interpreted as an incidence or definitive effect estimate for a unified diagnostic entity.

### 2.7 Risk-of-bias assessment and certainty of evidence

Randomized trials were appraised with the five domains of Cochrane RoB 2: randomization, deviations from intended interventions, missing outcome data, outcome measurement, and selection of the reported result (40). Assessments were outcome-specific and followed the RoB 2 signaling questions and domain-level decision rules. Two reviewers were involved in the RoB 2 and GRADE assessments: one reviewer performed the initial assessments, and a second reviewer independently checked all domain-level risk-of-bias judgments and outcome-level certainty ratings against the source reports and methodological guidance. Any disagreements were resolved through discussion and consensus. Particular attention was paid to allocation concealment, sham credibility, participant and assessor masking, attrition, repeated cognitive testing, registration, and prespecified analyses. Potential unblinding was considered under the deviations-from-intended-interventions domain when sensory differences between active and sham stimulation could plausibly have altered participant behavior, adherence, co-interventions, or other deviations from the assigned intervention. It was considered separately under the outcome-measurement domain when participants or outcome assessors could have inferred treatment allocation and the cognitive or delirium assessment was potentially susceptible to such knowledge. Physiological inertness of a sham condition was therefore not treated as equivalent to successful blinding. Detailed signaling-question responses, supporting trial information, and the rationale linking domain-level judgments to the overall RoB 2 assessment are provided in Supplementary Table S4. Certainty was evaluated with GRADE across risk of bias, inconsistency, indirectness, imprecision, and publication bias (41). For imprecision, we considered the total information size, number of contributing studies and events, width and position of the 95% confidence interval relative to the null, and availability of variance estimates. Because no prespecified or clearly established minimal important effect threshold was available for the perioperative taVNS outcomes evaluated in this review, we did not impose an arbitrary effect-size cutoff. Certainty was downgraded for imprecision when the available information size was limited and/or the confidence interval remained compatible with materially different clinical interpretations, or when variance information was incomplete. Indirectness was assessed according to the correspondence between the analyzed outcome construct and the prespecified clinical outcome of interest. Complete outcome-specific GRADE profiles and downgrade rationales are provided in Supplementary Tables S5a, S5b. Because fewer than 10 studies were available for any outcome, funnel plots and small-study-effect tests were not performed.

### 2.8 Statistical synthesis

Two trials with compatible binary event data were included in a *post hoc* exploratory cross-construct synthesis. Although the trials used different diagnostic frameworks—one assessing dNCR and the other investigator-defined traditional POCD—both reported binary measures of postoperative cognitive deterioration during the first postoperative week and addressed a similar clinical question regarding the effect of perioperative taVNS on early postoperative cognitive outcomes. Because these constructs were clinically related but not equivalent, the synthesis was undertaken only to examine concordance in the direction and approximate magnitude of treatment effects across the two outcomes, rather than to estimate the incidence or definitive treatment effect of a single standardized cognitive endpoint. This cross-construct synthesis was not prespecified in the PROSPERO registration and was introduced after study selection as an explicitly *post hoc*, hypothesis-generating analysis.

Study-level risk ratios and 95% confidence intervals were calculated on the log-risk-ratio scale using inverse-variance methods. Because only two studies were available and the synthesis was explicitly exploratory, the common-effect estimate was retained only as a descriptive inverse-variance summary of the two observed study estimates and was not interpreted as evidence that the studies shared a common underlying clinical effect. Between-study variance was estimated using the Paule–Mandel method by solving Q(τ^2^) = k − 1 with weights 1/(vᵢ + τ^2^); when Q(0) ≤ k − 1, τ^2^ was set to zero. A Hartung–Knapp sensitivity interval was calculated using the Paule–Mandel weights, with the variance scaling factor defined as QPM/(k − 1) and inference based on the Student t distribution with k − 1 degrees of freedom. No *ad hoc* variance-floor or modified Hartung–Knapp adjustment was applied when the Hartung–Knapp variance estimate was smaller than the conventional random-effects variance. All calculations were implemented in Python 3.12.13 using standard-library code. Cochran’s Q, I^2^, and τ^2^ were reported descriptively. Because only two studies contributed, both between-study heterogeneity and random-effects uncertainty were estimated very imprecisely; therefore, an I^2^ of 0% was not interpreted as evidence of clinical homogeneity. No continuity correction was required because all event cells were non-zero. POD and longitudinal MoCA outcomes were analyzed separately because they represented distinct clinical constructs and assessment frameworks.

### 2.9 Identification and narrative synthesis of mechanistic and translational evidence

Mechanistic and translational evidence was identified separately from the systematic clinical-efficacy review through four complementary routes: records retrieved during the clinical search, key reviews in taVNS and vagus-nerve physiology, backward and forward citation tracing, and topic-specific searches updated through 5 August 2026. The thematic searches combined taVNS or vagus-nerve-stimulation terms with concepts related to inflammation and the cholinergic anti-inflammatory pathway, autonomic regulation and heart-rate variability, blood–brain barrier and neurovascular-unit function, glial responses, sleep, pain, cognition, neuroimaging, stimulation parameters, sham design, safety, and translational trial methodology.

Sources were retained for detailed discussion when they informed at least one of the following narrative domains: (1) anatomical or physiological plausibility of auricular vagal stimulation; (2) perioperative or surgery-related biological mechanisms; (3) neural or physiological target engagement; (4) stimulation dose, timing, or parameter selection; (5) comparator and sham design; (6) safety and tolerability; or (7) translational implications for future perioperative trials. In selecting sources for detailed discussion, priority was given to studies that were more directly relevant to the perioperative cognitive question and to original studies supporting specific mechanistic claims. Systematic and narrative reviews were used primarily for context and citation tracing.

Evidence was organized according to its directness to the perioperative review question into four categories: perioperative human evidence, surgery-related preclinical evidence, non-perioperative patient evidence, and healthy-participant target-engagement evidence. Perioperative human studies were considered the most directly applicable to the clinical question. Surgery-related preclinical studies were used to support biological plausibility but not to establish clinical mediation. Non-perioperative patient studies and healthy-participant studies were used primarily to inform target engagement, physiological effects, dose selection, or mechanistic plausibility and were not treated as direct evidence of perioperative cognitive benefit.

This component was a structured narrative synthesis rather than a second systematic review. It was therefore not included in the clinical PRISMA counts, no quantitative meta-analysis of mechanistic studies was performed, and RoB 2 or GRADE was not applied across the entire mechanistic literature. The narrative evidence library comprised 132 individually verified sources. Yuhan Meng organized the broader evidence library and classified sources according to their relevance and directness to the review question, and Ruyi Xia subsequently cross-checked source relevance and evidence classification. The search was intended to provide structured coverage of the major mechanistic and translational domains rather than exhaustive retrieval of every mechanistic publication. Mechanistic conclusions were interpreted according to the directness of the underlying evidence, with preclinical and non-perioperative findings used to support biological plausibility rather than establish causal mediation or clinical efficacy.

## 3 Results

### 3.1 Study selection

The database searches identified 978 records: 153 from PubMed/MEDLINE, 450 from Embase, 126 from Web of Science, and 249 from CENTRAL. After removal of 342 duplicates, 636 unique records underwent title-and-abstract screening. A total of 632 records were excluded. Four reports were retrieved and assessed in full, none was excluded after full-text assessment, and four distinct randomized clinical trials were included. The detailed PRISMA 2020 study-selection flow is presented in Figure 1.

**Figure 1:**
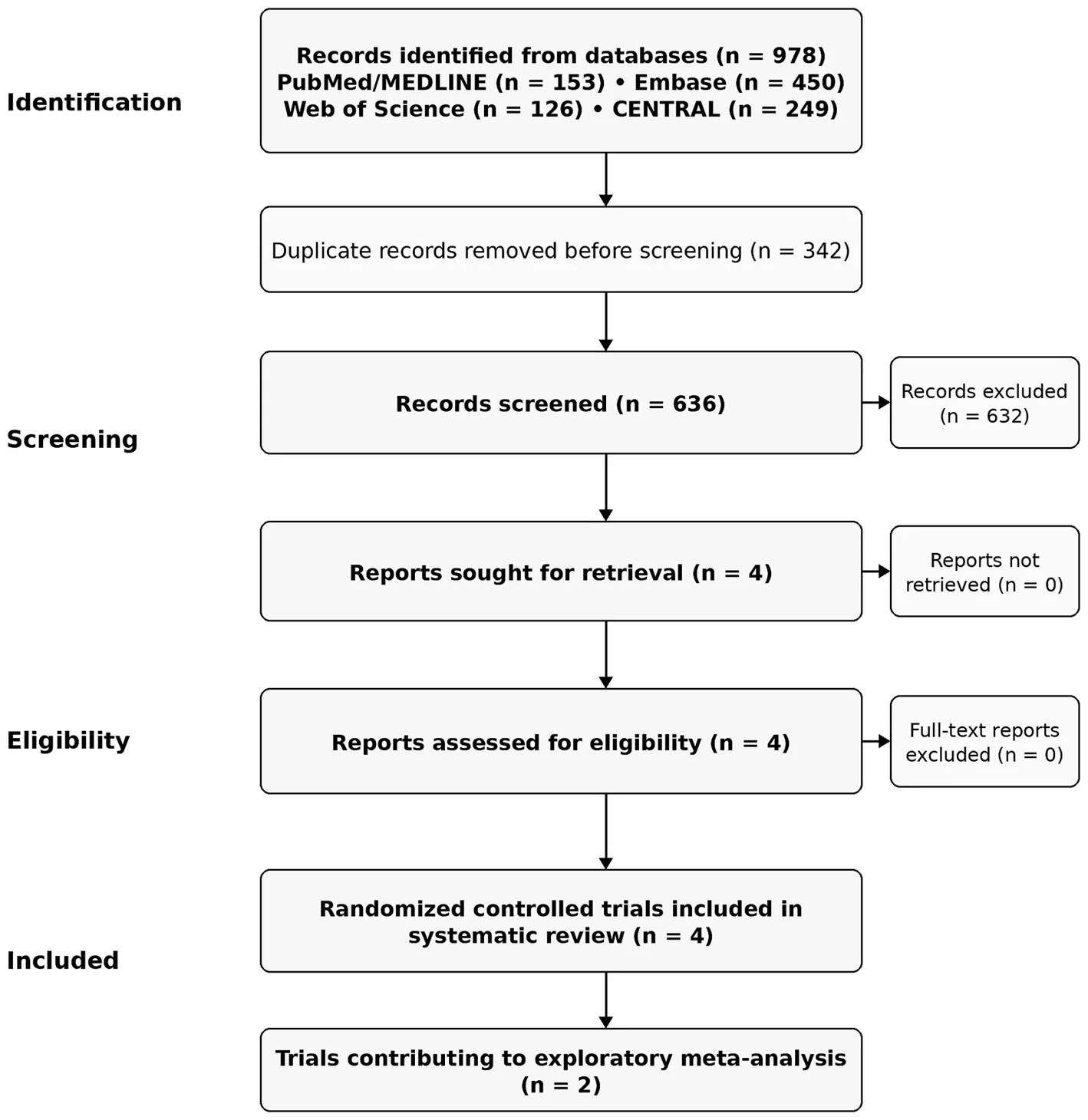
PRISMA 2020 flow diagram for identification and selection of direct perioperative randomized clinical evidence. Mechanistic and translational sources used in the narrative synthesis were not included in these counts. Protocols and ongoing studies were described separately and were not counted as completed efficacy trials.

### 3.2 Characteristics of included randomized trials

The four trials were published in 2022 or 2026 and enrolled older adults undergoing total joint arthroplasty, major non-cardiac surgery, or gastrointestinal tumor surgery (42–45). In total, 480 participants were enrolled or randomized and 469 contributed to reported analyses. Cognitive endpoints included dNCR defined with a neuropsychological battery, investigator-defined early POCD, POD assessed with CAM/CAM-ICU, and serial MoCA scores through 3 months. Study-level characteristics are summarized in Table 1.

**Table 1:** Design, population, cognitive outcomes, and principal findings of the four included randomized trials.

Study/setting/surgery	Randomized/analyzed n	Participant characteristics	Intervention/comparator	Outcome definition/time point	Principal finding	Safety/limitations
Zhou et al., 2022 (43). Single-center trial, Third Hospital of Hebei Medical University; total joint arthroplasty.	124 randomized (62/62); 119 completed week-1 neuropsychological testing (60/59).	taVNS vs. sham: age 74.5 ± 5.6 vs. 74.2 ± 5.2 y; women 33/62 vs. 34/62; BMI 23.9 ± 3.2 vs. 24.2 ± 2.8; education median 5 (IQR 3–8) vs. 5 (3–6); MMSE 28.1 ± 1.9 vs. 28.0 ± 1.6. ASA I/II/III 11/41/10 vs. 12/38/12. Hypertension 46/62 vs. 48/62; diabetes 22/62 vs. 20/62; prior cerebral infarction 20/62 vs. 16/62; coronary artery disease 16/62 vs. 17/62.	Left cymba-conchae taVNS vs. left-ear sham at earlobe and tail of helix; further details in Table 2.	dNCR at postoperative week 1, defined using a neuropsychological test battery and a > 1.96 SD decline relative to matched nonsurgical controls to account for practice effects.	dNCR: 6/60 vs. 16/59; *p* = 0.016.	Table 3 reports perioperative bradycardia, hypotension, tachycardia, hypertension, and PONV by group; these events were not attributed to stimulation. Authors reported no stimulation-related pain, itching, or headache, but group-specific counts were not given. Allocation concealment was NR; 5 lacked week-1 cognitive data.
Shi et al., 2026 (44). Prospective multicenter trial at three hospitals; adults aged 65–85 undergoing major non-cardiac surgery (>1 h).	150 randomized (75/75); all 150 completed follow-up.	Sham vs. active taVNS: age median 72 (IQR 70–74) vs. 73 (70–77); sex 45/30 vs. 51/24 male/female (counts as reported); BMI 23.3 ± 3.3 vs. 23.4 ± 3.1; MMSE 26 (23–28) vs. 26 (23–29). ASA II/III 43/32 vs. 29/46. Diabetes 14/15; hypertension 37/43; prior cerebral infarction 9/12; coronary artery disease 5/3.	Active left cymba-conchae stimulation vs. left-earlobe stimulation using the same program; detailed parameters in Table 2.	POD within postoperative days 1–3, assessed twice daily for 3 days using CAM or CAM-ICU (six planned assessments).	POD: 9/75 vs. 19/75; RR 0.47 (95% CI 0.23–0.98), *p* = 0.036.	AE ascertainment methods were described but group-specific AE counts/results were not reported. Stimulation administrators knew allocation; outcome assessors and clinical staff were masked. One baseline ASA imbalance (*p* = 0.022) was considered compatible with chance; no formal blinding-success assessment.
Li et al., 2026 (42). Single-center trial at Jiaxing Second Hospital; elective gastrointestinal tumor surgery.	103 randomized (51/52); 101 in primary analysis (49/52). Two active-arm participants withdrew: procedure cancellation and refusal of postoperative stimulation.	Primary-analysis population, active vs. sham: age median 70 (IQR 67–75) vs. 68 (67–71.25); male/female 32/17 vs. 29/23; ASA II/III 39/10 vs. 43/9; baseline MoCA categories 23–<26: 18/49 vs. 24/52, and ≥26: 31/49 vs. 28/52. Stomach/bowel tumor 10/39 vs. 19/33; hypertension 27/49 vs. 31/52; diabetes 10/49 vs. 12/52; cardiovascular disease 2/49 vs. 3/52; respiratory disease 5/49 vs. 6/52.	Auricular taVNS vs. same reported operation parameters with 0 mA; the sham site is not separately specified. Detailed regimen in Table 2.	Investigator-defined early POCD: ≥2-point MoCA decrease from baseline to POD7; MoCA was also assessed on POD1 and POD3. This is not a consensus dNCR diagnosis.	POCD: 5/49 vs. 17/52; *p* = 0.006. POD incidence was not significantly different. PONV was 3/49 vs. 12/52 as a clinical secondary outcome, not an attributed device AE.	Structured AE definitions, ascertainment, and group-specific device-related AE counts were NR. Cognitive assessors/data collectors were blinded; participant and intervention-provider masking were not described. Registration was reported as retrospective; the article also states recruitment began after registration, an internal timing inconsistency.
Ma et al., 2026 (45). Prospective single-center trial at Shanghai Pudong New Area People’s Hospital; elective laparoscopic gastrointestinal tumor surgery.	103 randomized (53/50); 99 complete-case analysis (49/50). Four active-arm participants withdrew consent after randomization and before intervention; an ITT multiple-imputation sensitivity analysis included all 103.	Complete-case population, active vs. control: age 70.8 ± 3.8 vs. 72.8 ± 5.4 y; male/female 25/24 vs. 26/24; BMI 33.5 ± 3.1 vs. 32.2 ± 3.9; baseline MoCA 25.6 ± 1.9 vs. 26.1 ± 2.1. ASA and comorbidity distributions by group were NR in the published baseline table; eligibility criteria are not substituted for baseline data.	Left cymba/cavum-conchae taVNS vs. earlobe control with identical device appearance/interface and no current; detailed parameters in Table 2.	Serial MoCA at baseline, POD1, POD7, postoperative month 1, and month 3; a screening-score trajectory, not a standardized PND diagnosis.	Adjusted between-group differences favored taVNS by +2.06 points at month 1 and +2.97 at month 3 (both *p* < 0.001); complete CIs/SE for adjusted differences were unavailable. MI sensitivity in 103 yielded consistent inferences.	AEs: 2/49 (4.1%) vs. 2/50 (4.0%), all described as mild local auricular erythema/transient tingling; no serious AEs observed. Exact event type by arm NR. Single-center, modest sample; assessor masking reported, intervention-provider masking NR; sequence-generation method NR.

For Shi et al. (44) sex counts are reproduced as reported; the published percentages for the active arm were inconsistent with the stated group size and were therefore not reproduced.

### 3.3 taVNS regimens and comparator characteristics

Zhou and Shi delivered a single surgery-linked session beginning before anesthetic induction; Li used five daily 30-min sessions from the day before surgery through postoperative day 4; and Ma prescribed 30-min sessions twice daily over seven consecutive days, with the article also describing a window from one day preoperatively through postoperative day 7. Frequency, pulse width, current titration, duty cycle, laterality, and comparator conditions differed across trials. Numerical current was not reported for Zhou or Shi, and the delivered current was not reported for Ma; Table 2 distinguishes reported settings from actual delivered values. These differences limit dose–response inference and indicate that study effects do not represent one standardized taVNS intervention or equivalent comparator.

**Table 2:** taVNS protocol and comparator characteristics.

Study	Device/electrode geometry	Active site/laterality/rationale	Waveform/frequency/pulse width	Programmed or delivered current/impedance monitoring	Duty cycle/session duration/timing/number of sessions	Sham site/program/blinding	Adherence/registration/key interpretive issue
Zhou 2022 (43)	Roscoe TENS 7000; custom-developed concha electrode plus clip behind the earlobe; contact area/geometry NR.	Left cymba conchae; laterality rationale NR.	Modulation mode; 10 Hz; 300 μs; waveform shape NR.	Amplitude set to maximum tolerated without pain; numeric programmed or delivered mA NR. Impedance measurement/ monitoring NR.	Started 1 h before anesthetic induction and continued until surgery ended; session duration depended on surgery; duty cycle and session count NR.	Left earlobe and tail of helix; same general perioperative timing. Sham electrical output/current and sensory matching NR. Patients/investigators described as blinded; nurse placed electrodes; no formal blinding-success assessment.	Three participants did not receive assigned intervention (two early terminations, one combined IV–inhalation anesthesia); reasons for early termination NR. Week-1 testing: 119/124. ChiCTR2100044905.
Shi 2026 (44)	tVNS501 (Rishena, Changzhou); two 4.5-mm Ag/AgCl electrodes; contact area and detailed geometry NR.	Left cymba conchae. Authors state left side was selected to avoid potential cardiac effects attributed to right auricular vagal projections.	Biphasic pulses; 40 Hz; 500 μs; 50 s ON**/**10 s OFF.	Raised from minimum until prickling and adjusted to tolerable, painless intensity; numerical mA and actual delivered output NR. Gel applied to reduce impedance; impedance was not measured/reported.	One surgery-dependent session, started 5 min before induction and continued to end of surgery; fixed duration NR.	Left earlobe; same parameters and duration, with site as stated group difference. Participant masking implied by double-blind design; administrators unblinded; clinical staff and assessors masked; no formal blinding-success assessment.	All 150 completed; session-level adherence NR. ChiCTR2400090660. Earlobe stimulation may not be physiologically inert.
Li 2026 (42)	Changzhou Ruishen’an Medical Instrument Co., Ltd., Ruishen’an tVNS501; electrode type/geometry/contact area NR.	Active cymba of auricular concha; laterality and rationale NR.	Alternating 4/20-Hz expansion waves (4 Hz for 3 s, 20 Hz for 7 s); pulse width 200 μs ± 10%.	Intensity described as maximum tolerance, 0.8–1.5 mA; individual delivered value NR. Impedance monitoring NR.	30 min once daily at 17:00 on preoperative day 1 and POD1–POD4; five planned sessions; duty cycle NR.	0 mA with other reported operation parameters the same; sham site not separately specified. Outcome assessors/data collectors masked; participant/provider masking and formal blinding success NR.	Two active-arm withdrawals (procedure cancellation; refusal of postoperative stimulation); session-level adherence NR. ChiCTR2500115495; registration reported as retrospective.
Ma 2026 (45)	taVNS device with single-use auricular electrode patches; manufacturer/model, electrode type/geometry/contact area NR.	Left cymba and cavum conchae; rationale for laterality NR.	30 Hz; 100 μs; waveform shape NR; 10 s ON**/**10 s OFF.	Individual staircase binary-search titration to comfortable intensity; numeric actual/programmed current NR. Impedance monitoring NR.	30 min/session, twice daily for 7 consecutive days; article states start 1 day preoperatively through POD7, so exact calendar-day/session count is internally unclear.	Earlobe; identical appearance/ interface, no current after activation. Double-blind design stated; assessors explicitly unaware; participant/provider-specific masking and formal blinding-success assessment NR.	Four active-arm participants withdrew before receiving intervention; completed session count/adherence among treated participants NR. ChiCTR2400094874.

### 3.4 *Post hoc* exploratory cross-construct synthesis of early postoperative cognitive decline

Two trials reported binary measures of early postoperative cognitive deterioration but used different diagnostic constructs. Zhou et al. reported dNCR at postoperative week 1 in 6 of 60 participants assigned to taVNS and 16 of 59 controls (43), whereas Li et al. reported investigator-defined traditional POCD during postoperative days 1–7 in 5 of 49 participants assigned to taVNS and 17 of 52 controls (42). Because both studies addressed early postoperative cognitive deterioration within broadly comparable perioperative settings but did not use equivalent diagnostic definitions, they were combined only in a *post hoc* exploratory cross-construct synthesis. The study-specific estimates were therefore regarded as the primary basis for interpretation, whereas the pooled estimate was considered only a secondary, hypothesis-generating summary.

The exploratory pooled RR was 0.34 (95% CI 0.18–0.64; *p* = 0.0008), with Q = 0.07, I^2^ = 0%, and τ^2^ = 0 (Figure 2). Because only two studies contributed data and only 44 events were observed in total, both between-study heterogeneity and random-effects uncertainty were estimated very imprecisely. Accordingly, I^2^ = 0% should not be interpreted as evidence of clinical homogeneity between the two outcome constructs. Because the Paule–Mandel estimate of between-study variance was τ^2^ = 0, the common-effect and Paule–Mandel random-effects models yielded the same point estimate and confidence interval to two decimal places (RR 0.34, 95% CI 0.18–0.64). This numerical agreement should not be interpreted as evidence that the studies shared a common underlying clinical effect. Hartung–Knapp sensitivity analysis yielded a wider 95% CI of 0.12–0.98 while preserving the direction of effect.

**Figure 2:**
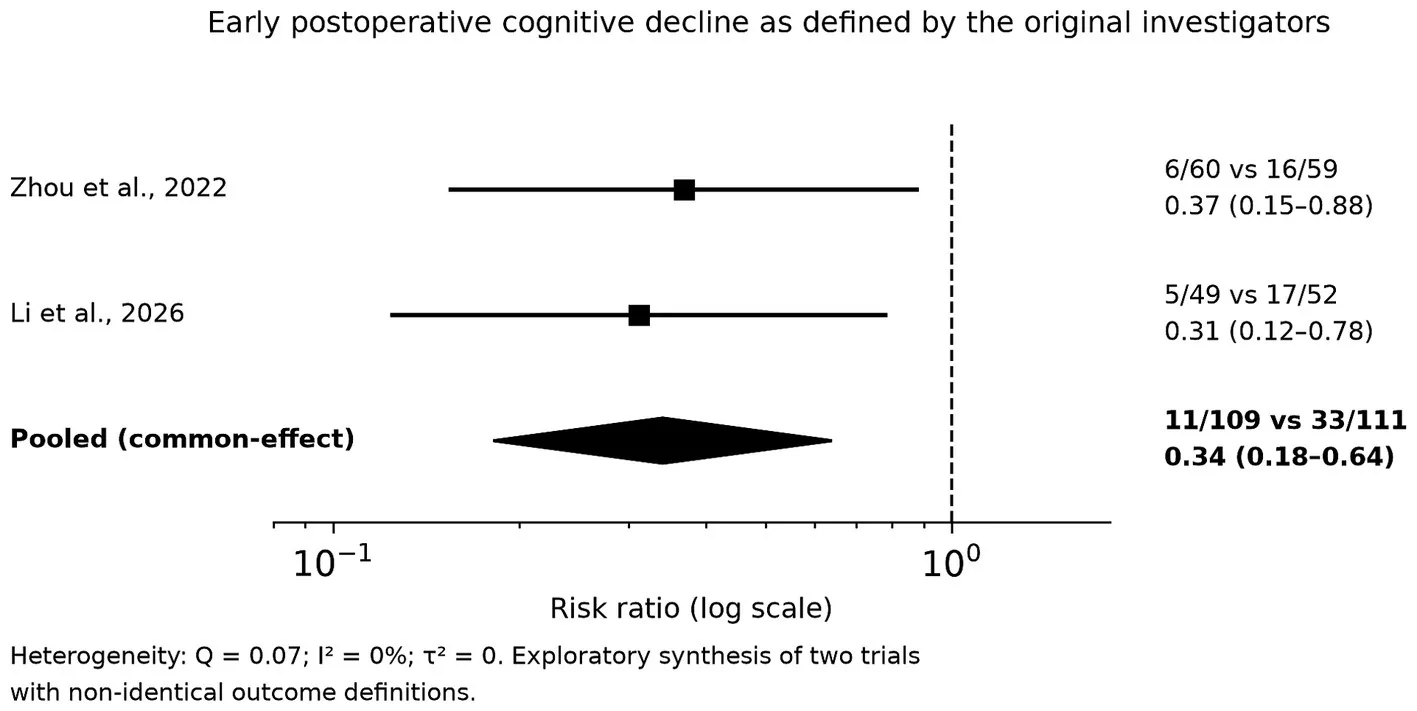
*Post hoc* exploratory cross-construct synthesis of two early postoperative cognitive-decline outcomes. Zhou et al. (43) assessed dNCR, whereas Li et al. (42) assessed investigator-defined traditional POCD. The pooled estimate summarizes concordance in treatment effects across related but non-identical constructs and should not be interpreted as an effect estimate for a single standardized outcome.

The pooled RR should therefore be interpreted only as an exploratory summary of concordant treatment effects across two clinically related but non-identical early postoperative cognitive-decline constructs, rather than as a definitive summary effect for a single standardized clinical outcome.

### 3.5 Postoperative delirium

POD was evaluated as a primary endpoint in the multicenter trial by Shi et al. (44). During postoperative days 1–3, POD occurred in 9/75 participants in the taVNS group and 19/75 in the sham group, yielding RR 0.47 (95% CI 0.23–0.98; *p* = 0.036). This result was not combined with dNCR or the investigator-defined traditional POCD outcome because delirium is a distinct acute neuropsychiatric syndrome with a different assessment framework and time course. The authors also reported improved postoperative sleep quality and a mediation analysis suggesting that sleep improvement may have accounted for part of the observed association. Only one completed POD trial was available.

### 3.6 Longitudinal cognitive outcomes, biomarkers, and safety

Ma et al. measured MoCA at baseline and postoperative days 1 and 7 and months 1 and 3 (45). Of 103 randomized participants, four assigned to taVNS withdrew consent after allocation but before receiving intervention; the complete-case analysis included 99 participants (49 taVNS, 50 control). The authors also reported an intention-to-treat multiple-imputation sensitivity analysis including all 103 participants, with consistent inferences. Repeated-measures analyses found adjusted between-group differences favoring taVNS of +2.06 points at month 1 and +2.97 points at month 3 (both *p* < 0.001); complete confidence intervals or standard errors for these adjusted differences were not available in the article. Accordingly, the trajectory is described rather than pooled. Serial MoCA scores are not equivalent to a standardized dNCR or postoperative NCD diagnosis, and statistical significance does not establish clinical importance.

Biomarker findings differed across the four trials. Zhou et al. reported lower postoperative AChE/BChE activity and smaller increases in IL-6, HMGB1, and S100β with taVNS than sham; the between-group change in TNF-α was not significant (43). Li et al. reported lower plasma S100β and IL-6 on postoperative days 1–2 (42). Ma et al. reported lower IL-1β and S100β on postoperative day 7 (45). Shi et al. found no between-group differences in CRP or neutrophil-to-lymphocyte ratio; the trial reported improved postoperative sleep and a statistical mediation analysis, which does not by itself establish causal mediation (44). These peripheral and behavioral changes are parallel observations, not proof of central target engagement or mechanism. Perioperative safety reporting was heterogeneous. Zhou et al. reported group-level bradycardia, hypotension, tachycardia, hypertension, and PONV counts, but did not attribute these events to stimulation; the authors also stated that no stimulation-related pain, itching, or headache occurred, without group-specific counts (43). Shi et al. specified self-report, medical-record review, and investigator observation, but did not report group-specific AE results (44). Li et al. did not provide a structured device-AE assessment; its PONV outcome (3/49 vs. 12/52) is not treated as a stimulation-related AE (42). Ma et al. reported AEs in 2/49 (4.1%) and 2/50 (4.0%), all mild local auricular erythema or transient tingling overall, and no serious AEs; local-event type by group was not reported (45). Unreported counts are not interpreted as zero, and these small trials cannot characterize uncommon harms.

### 3.7 Risk of bias

Outcome-specific RoB 2 judgments are summarized in Figure 3, with full signaling-question responses and primary-source locations in Supplementary Table S4. Zhou was rated Some concerns in D1, D2, and D5 and Low in D3–D4; Shi was Low in D1–D4 and Some concerns in D5; Li was Some concerns in D1, D2, and D5 and Low in D3–D4; Ma was Some concerns in D1, D2, and D5 and Low in D3–D4. All four overall judgments were Some concerns, with no domain rated High. For Li, the article’s description of retrospective registration conflicts with its statement that recruitment began after registration; the discrepancy and absence of a dated analysis plan informed D5.

**Figure 3:**
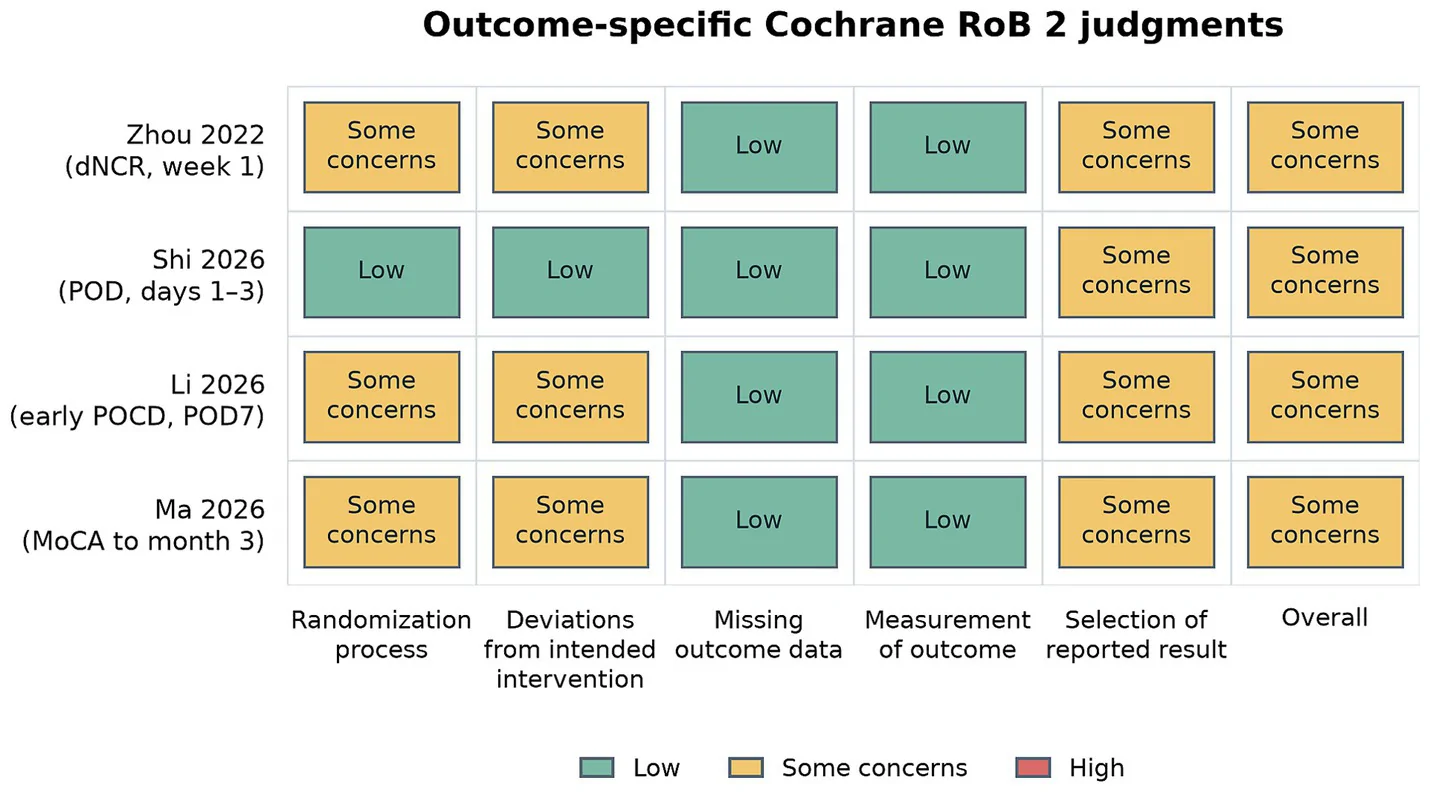
Outcome-specific RoB 2 domain judgments for the principal clinical outcome assessed in each included trial.

### 3.8 Certainty of evidence

Certainty for the *post hoc* exploratory cross-construct synthesis of dNCR and investigator-defined traditional POCD was rated very low because both contributing trials had outcome-specific RoB 2 judgments of Some concerns, the outcome constructs are not diagnostically equivalent, and only 44 events were observed among 220 participants. Inconsistency was not downgraded, but its assessment is limited by only two studies; I^2^ = 0% does not establish clinical homogeneity. Certainty was low for POD because the single contributing trial had Some concerns overall and only 28 events. Certainty was very low for the longitudinal MoCA trajectory because of Some concerns in RoB 2, indirectness relative to a standardized PND diagnosis, and unavailable confidence intervals/standard errors for the adjusted month-1 and month-3 differences. The final grades are unchanged; detailed profiles are in Table 3 and Supplementary Tables S5a, S5b.

**Table 3:** GRADE summary of certainty for principal cognitive outcomes.

Outcome	Studies/participants	Effect estimate	Certainty	Main reasons for downgrading
*Post hoc* exploratory cross-construct synthesis of dNCR and investigator-defined traditional POCD	2 RCTs**/**220	RR 0.34 (95% CI 0.18–0.64)	Very low	Risk of bias; indirectness due to the combination of related but non-equivalent dNCR and investigator-defined traditional POCD constructs; and imprecision due to sparse events and limited information size.
Postoperative delirium	1 RCT**/**150	RR 0.47 (95% CI 0.23–0.98)	Low	Single trial; imprecision; some concerns regarding selective reporting.
Longitudinal MoCA trajectory	1 RCT**/**99	MD + 2.06 at month 1 and +2.97 at month 3	Very low	Risk of bias; indirectness; incomplete variance data; single small trial and repeated-testing effects.

The pooled estimate for dNCR and investigator-defined traditional POCD represents a post hoc exploratory cross-construct synthesis. These outcomes were not considered diagnostically equivalent, and the pooled RR should not be interpreted as the incidence or treatment effect of a single standardized cognitive endpoint.

### 3.9 Published protocols and ongoing trials

Published protocols and registry records describe a larger emerging research landscape, including the VNSTAR trial, total-knee-arthroplasty protocols, hip-fracture surgery, and vulnerable-brain-function populations (4, 5, 46, 47). These studies inform planned stimulation regimens, sham conditions, POD assessments, biomarkers, and longer-term outcomes but do not constitute efficacy evidence. Their main design features and methodological considerations are summarized in Table 4.

**Table 4:** Published protocols and ongoing perioperative taVNS trials.

Study	Design and planned population	taVNS regimen and control	Planned outcomes	Main methodological considerations
Wu et al. (4) (NCT06421077)	Multicenter, double-blind, parallel randomized trial; planned enrollment of 1,776 patients aged ≥65 years undergoing elective surgery, excluding neurosurgery and cardiac surgery.	Standard taVNS: 25 Hz, 250 μs, maximum painless tolerated intensity; active low-frequency control: 1 Hz, 250 μs. Repeated delivery before, during, and early after surgery.	POD within 5 days, assessed twice daily with 3D-CAM/CAM-ICU; 90-day cognitive and functional outcomes.	Protocol only; the low-frequency control may have biological effects; heterogeneous surgical and anesthetic procedures.
Liao et al. (5) (registration not reported in protocol publication)	Four-center, randomized, double-blind, sham-controlled trial; planned enrollment of 1,448 patients aged 65–80 years undergoing elective total knee arthroplasty under general anesthesia.	taVNS: cymba conchae and tragus, 25 Hz, 250 μs; sham: earlobe and antihelix with the same frequency and pulse width. One session on the afternoon before surgery and one on the morning before anesthesia, at the maximum painless tolerated intensity.	POD within 72 h; inflammatory mediators, HRV, sleep, pain, and cognitive trajectories at 1, 3, 6, and 12 months.	Protocol only; restricted to total knee arthroplasty; whether only two preoperative sessions achieve stable target engagement is uncertain.
Zhou et al. (46) (ChiCTR2400085508)	Single-center, randomized, double-blind, sham-controlled trial; planned enrollment of 154 older adults undergoing hip-fracture surgery.	taVNS: 10 Hz, 300 μs; current increased until tingling and then set slightly below that threshold; from 1 h before induction to the end of surgery. Sham: effective current discontinued after the sensory threshold was reached.	POD assessed with CAM-ICU, cognition at 7 days, cholinesterases, TNF-α, IL-1β, IL-6, and safety.	Protocol only; single center; complex clinical status and time-sensitive surgery; current cessation may compromise blinding; limited long-term outcomes.
Hao et al. (47) (ITMCTR2025002263)	Two-center, double-blind, randomized trial; planned enrollment of 148 patients aged ≥60 years with non-acute vulnerable brain function undergoing abdominal surgery.	Bilateral stimulation for 30 min; alternating 4 Hz (5 s) and 20 Hz (10 s). Control: active stimulation of a non-vagal auricular region.	POD within 5 days; delirium severity, biomarkers, delayed cognitive decline, pain, gastrointestinal function, complications, and economic outcomes.	Protocol only; the specific definition of vulnerable brain function requires external validation; heterogeneous participants and comorbidities; inertness and blinding validity of the active control require assessment.

Protocols describe planned research and do not constitute efficacy evidence.

## 4 Mechanistic and translational synthesis

### 4.1 Neuroimmune pathophysiology of PND

PND is not a single disease entity but a diagnostic framework encompassing different time windows and clinical phenotypes (1–3). POD usually develops within hours to days after surgery and is characterized by an acute, fluctuating disturbance of attention and awareness. dNCR refers to objective, baseline-adjusted cognitive decline within 30 days after surgery, whereas postoperative NCD describes cognitive impairment persisting from 30 days to 12 months and meeting diagnostic criteria for a neurocognitive disorder (3). Affected domains may include learning and memory, executive function, attention, processing speed, and visuospatial function (48, 49). Clinical studies should use a preoperative baseline and a neuropsychological test battery covering multiple cognitive domains. A single decline in the Montreal Cognitive Assessment (MoCA) score may serve as a screening or supportive measure but cannot replace a formal PND diagnosis (6). Preoperative cognitive impairment is an important predictor of adverse postoperative cognitive outcomes, while persistent postoperative deterioration is associated with longer hospitalization, long-term functional decline, and increased mortality (6, 7). POD, dNCR, and postoperative NCD should therefore be reported separately rather than grouped under the traditional POCD construct.

The initiating event in PND is commonly considered to be the systemic inflammatory response triggered by surgical injury, which may propagate to the central nervous system through a neuroimmune signaling cascade (8–10). Tissue injury releases DAMPs, among which high-mobility group box 1 (HMGB1) is an important pro-inflammatory mediator that initiates and amplifies peripheral inflammation (11). Circulating concentrations of interleukin-1β (IL-1β), IL-6, and TNF-α rise rapidly; these cytokines participate in local inflammation and enter the circulation to sustain systemic inflammatory signaling (11, 12). Peripheral monocytes and neutrophils are activated and recruited to injured tissues and the central nervous system, further increasing inflammatory-mediator release (12). This systemic inflammatory response is particularly prominent after cardiopulmonary bypass. Increases in circulating TNF-α and IL-6 may accompany region-specific changes in microglial morphology and function, suggesting dynamic coupling among the intensity of peripheral inflammation, the type of surgery, and the central immune response (13).

Surgery-related preclinical studies also implicate vagal-dependent mast-cell signaling and synaptic NMDA-receptor dysfunction in postoperative cognitive change (48–51).

Blood–brain barrier (BBB) dysfunction is a critical bridge between perioperative systemic inflammation and central neuroinflammation (14, 15, 52). Surgery and anesthesia may injure cerebral microvascular endothelial cells, reduce the expression of tight-junction proteins such as ZO-1, claudin-5, and occludin, and disrupt adherens-junction organization, thereby compromising BBB integrity (14, 53, 54). Endothelial metabolic reprogramming and mitochondrial dysfunction also contribute to barrier injury (55, 56). Inflammatory mediators such as IL-1β and TNF-α can directly act on cerebral microvascular endothelium, disrupt tight-junction architecture, and markedly increase BBB permeability (54).

As BBB permeability increases, circulating pro-inflammatory cytokines, activated immune cells, fibrinogen, and other potentially neurotoxic components gain access to the brain parenchyma (11, 16, 17). Fibrinogen binds to CD11b on microglia and activates PI3K/Akt/RhoA signaling, promoting a neurotoxic microglial state and the release of pro-inflammatory mediators (16). Peripheral monocytes and neutrophils may also cross the injured BBB in response to chemotactic signals and further amplify neuroinflammation (12, 54). Once initiated, the central inflammatory response can become self-reinforcing: activated microglia and astrocytes release additional cytokines and matrix metalloproteinases such as MMP-9, which further damage tight-junction proteins; sustained barrier permeability then permits continued entry of peripheral inflammatory signals (18, 19). This reciprocal relationship between BBB dysfunction and neuroinflammation is central to PND progression and may be especially pronounced in older individuals because aging is accompanied by impaired barrier function and reduced immune-regulatory capacity (14, 17, 20).

When systemic inflammatory signals reach the central nervous system, microglia—the resident immune cells of the brain—become activated, representing a central event in neuroinflammation (21–23). Activated microglia may adopt pro-inflammatory or neurotoxic-associated states and release cytokines, chemokines, and reactive oxygen species, reinforcing the inflammatory cascade (18, 22, 24). The NLRP3 inflammasome has a key role: its assembly and activation can mediate pyroptosis and promote maturation and release of IL-1β and IL-18, thereby aggravating neuronal injury (21, 25). Astrocytes also participate in inflammatory amplification and interact with microglia to drive disease progression (23, 26, 57). Neuroinflammation directly impairs neuronal and synaptic function. Disrupted synaptic plasticity is a major substrate of cognitive decline; inflammatory mediators disturb glutamatergic homeostasis, promote synaptic glutamate accumulation, and induce excitotoxicity and synaptic injury (58–60). Reduced expression of postsynaptic density protein 95 and dysregulation of brain-derived neurotrophic factor/TrkB signaling further weaken synaptic structure and function (58, 61). These pathological changes ultimately impair learning, memory, and other cognitive domains, with marked deficits in hippocampus-dependent memory observed in animal models (18, 62).

Perioperative autonomic imbalance is another important mechanism that may promote neuroinflammation and cognitive impairment (27–29). Surgical stress and pain can produce excessive sympathetic activation together with reduced vagal tone, disrupting sympathovagal balance (30). Clinically, this may be reflected by reduced heart rate variability (HRV), indicating impaired cardiac autonomic regulation (30). Reduced vagal tone not only compromises cardiovascular stability but may also weaken the endogenous cholinergic anti-inflammatory pathway and disturb the inflammatory reflex (30).

Severe disruption of perioperative sleep rhythms is an independent risk factor for postoperative cognitive impairment (7, 31). Postoperative pain, environmental disturbance, and residual anesthetic effects alter sleep architecture, including reductions in slow-wave and rapid-eye-movement sleep (31). Sleep disturbance may promote neurocognitive impairment by activating neuroinflammation, increasing oxidative stress, impairing glymphatic clearance of metabolic waste such as β-amyloid, and reducing hippocampal neurotrophic factors and Sirtuin 1, thereby weakening neuronal repair and regeneration (31). Postoperative pain is itself a stressor, and the resulting increase in opioid exposure is also associated with delirium and cognitive decline; multimodal analgesia and opioid-minimizing strategies are therefore recommended as best practice (7).

Surgical stress also activates the hypothalamic–pituitary–adrenal (HPA) axis and causes excessive glucocorticoid release (32). Excess glucocorticoids can injure the neurovascular unit, compromise BBB integrity, and directly affect hippocampal neuronal structure and function, resulting in impaired memory-related synaptic plasticity (32). HPA-axis hyperactivation may act in concert with sympathetic excitation and vagal inhibition to further amplify systemic inflammation and autonomic imbalance (30, 32).

At the network level, PND is associated with abnormal functional connectivity across multiple cognition-related brain regions. Studies of multimodal brain-monitoring-guided anesthesia management suggest that preserving connectivity within cognitive networks, including the default mode network and prefrontal–hippocampal circuits, may help attenuate postoperative cognitive decline (33). Surgery and anesthesia may reduce fractional anisotropy in the prefrontal cortex, nucleus accumbens, and hippocampus, accompanied by neuronal degeneration and glial activation; these microstructural changes correlate with behavioral evidence of cognitive impairment (26). Sleep disturbance, pain, stress responses, and network dysconnectivity therefore interact to form a multidimensional pathological network influencing perioperative cognitive outcomes (see Figure 4).

**Figure 4:**
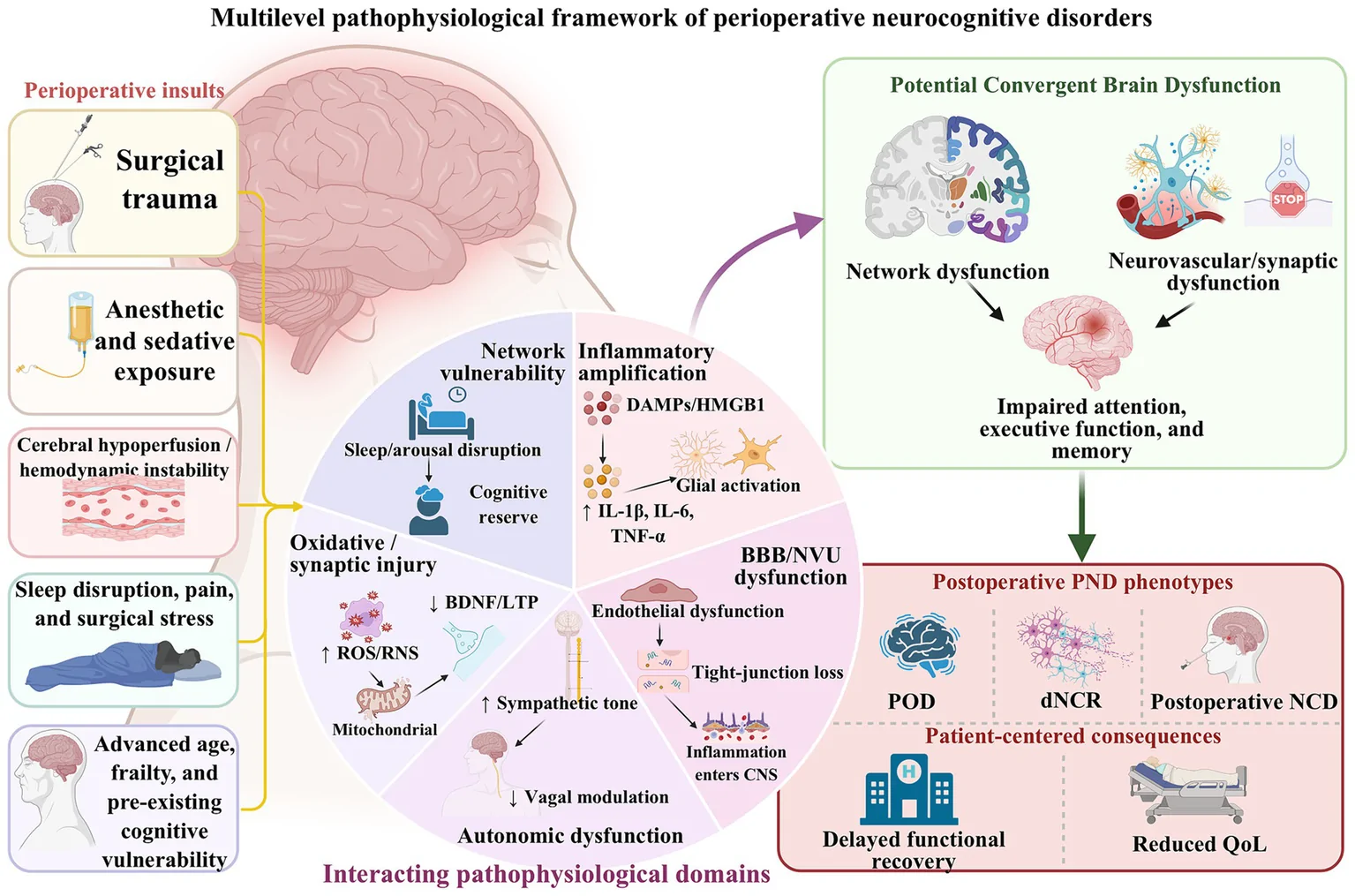
Multilevel pathophysiological framework of perioperative neurocognitive disorders. Perioperative insults are associated with interacting inflammatory, BBB/NVU, autonomic–neuroendocrine, oxidative–synaptic, and network-related processes that may contribute to postoperative brain dysfunction and distinct PND phenotypes. Arrows indicate proposed or conceptually linked relationships and should not be interpreted as establishing a linear or fully validated causal sequence in humans.

### 4.2 Anatomical and physiological basis of taVNS

The anatomical basis of taVNS is the distribution of the auricular branch of the vagus nerve (ABVN) in the external ear. The external auditory canal, cymba conchae, cavum conchae, and tragus are commonly used candidate sites, but the external ear also has overlapping trigeminal, facial, and cervical sensory innervation. Electrode position alone therefore cannot establish ABVN-specific recruitment (34, 35, 63).

When ABVN afferents are effectively engaged, their central projections are expected to reach the nucleus tractus solitarius (NTS) in the medulla (64). Animal studies have shown marked NTS activation after auricular stimulation; in one mouse study, the number of c-Fos-positive neurons in the medullary NTS increased 3.46-fold (65). Functional magnetic resonance imaging (fMRI) studies have likewise shown NTS activation in healthy participants during taVNS, with responses persisting after stimulation (66). From the NTS, vagal afferent signals are distributed to brain regions involved in cognition, emotion, and autonomic control. The NTS has direct and indirect projections to the locus coeruleus (LC), providing a potential route through which auricular stimulation may influence the LC–noradrenergic (LC–NE) system and processes related to attention, arousal, and stress responses (67–69). Ascending signals also reach the hypothalamus and participate in neuroendocrine and autonomic regulation (66). Limbic structures, including the amygdala and hippocampus, and prefrontal networks are additional targets. taVNS can modulate connectivity among the NTS, LC, limbic system, and prefrontal cortex (70), and can influence the default mode network, insula, cingulate cortex, and other key regions (71, 72). These anatomical connections provide a plausible neuroanatomical framework through which taVNS may influence neuroimmune and cognitive processes (36, 73). These observations are consistent with, but do not by themselves prove, selective vagal target engagement in perioperative patients (see Figure 5).

**Figure 5:**
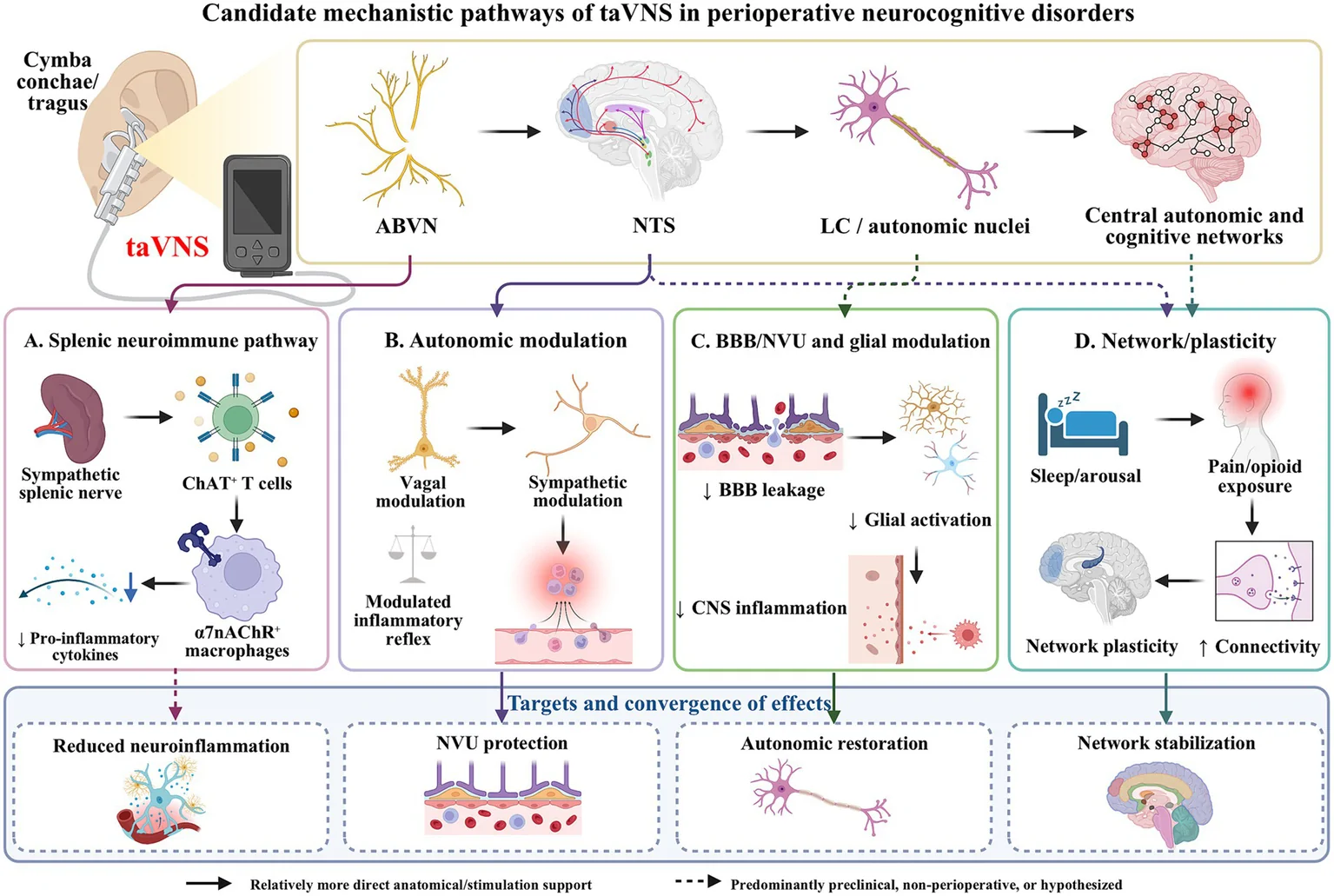
Candidate mechanistic pathways through which taVNS may influence perioperative neurocognitive disorders. Solid pathways indicate anatomical or stimulation relationships with relatively more direct supporting evidence, whereas dashed or dotted pathways indicate relationships that are predominantly hypothetical, preclinical, or extrapolated from non-perioperative evidence. Importantly, solid lines denote relative evidential directness rather than proof of pathway-specific mediation; none of the depicted pathways establishes a complete causal mechanism linking auricular stimulation to improved perioperative cognitive outcomes in humans.

### 4.3 Inflammatory reflex and cholinergic anti-inflammatory pathway

The inflammatory reflex comprises peripheral inflammatory sensing, brainstem integration, and descending immune regulation. taVNS is intended to recruit ABVN afferents that project mainly to the NTS and connect with the dorsal motor nucleus of the vagus, nucleus ambiguus, hypothalamus, and other autonomic nuclei (74–76). The CAP should not be simplified as direct release of acetylcholine from vagal terminals in the spleen onto macrophages. The more cautious model is that central integration modulates peripheral sympathetic and immune pathways, with the contribution of each anatomical relay in humans remaining incompletely resolved (77, 78). Animal studies show that taVNS can reduce TNF-α, IL-6, and IL-1β, and that α7nAChR blockade or deletion attenuates some effects (79–82). Perioperative biomarker changes occurring alongside cognitive outcomes do not establish CAP-mediated benefit; pathway-specific measurements and prespecified mediation analyses are required.

### 4.4 Splenic sympathetic–cholinergic immune-cell axis

The vagus nerve–splenic nerve–cholinergic immune-cell axis is the principal peripheral effector model of the CAP. Norepinephrine released from the sympathetic splenic nerve acts on β2-adrenoceptors on choline acetyltransferase-positive (ChAT^+^) T cells, stimulating acetylcholine release. Acetylcholine then binds α7nAChR on macrophages and suppresses NF-κB-related pro-inflammatory transcription and release of TNF-α, IL-1β, and IL-6 (35, 77, 78). This multisynaptic, multicellular circuit is more consistent with current neuroanatomical and immunophysiological evidence than a model of direct vagal innervation of splenic macrophages.

A randomized study in sepsis reported that taVNS reduced TNF-α and IL-1β, increased IL-4 and IL-10, and was accompanied by improved organ-function scores (83). Models of acute lung injury and gout have likewise shown reduced neutrophil infiltration, extracellular-trap formation, and tissue inflammation, with partial attenuation after α7nAChR blockade (82, 84). These findings support the anti-inflammatory feasibility of this axis but arise from disease contexts other than PND and therefore constitute indirect evidence. Whether perioperative taVNS limits transmission of peripheral inflammation to the brain through this immune axis requires direct testing in surgical models and patients, including measurements of ChAT^+^ T cells, splenic-nerve activity, and α7nAChR-related markers.

### 4.5 Autonomic regulation

Excessive perioperative sympathetic activation and reduced vagal regulation may promote inflammatory-reflex imbalance and organ dysfunction. Some studies report increases in the HRV measures SDNN, RMSSD, or pNN50 after taVNS and suggest that stimulation timing and baseline autonomic state may influence the response (85–88). A pooled analysis, however, did not show a consistent acute effect of taVNS on vagally mediated HRV (89). Current evidence therefore suggests that taVNS may influence sympathovagal regulation, but HRV changes alone cannot establish effective vagal target engagement. Perioperative improvements in HRV may support hemodynamic stability, yet causal links with cerebral perfusion, inflammatory suppression, and cognitive benefit remain unproven (5, 90). Future studies should combine HRV with blood-pressure variability, pupillary responses, electroencephalography or functional imaging, and inflammatory measures rather than treating it as a single mechanistic biomarker.

### 4.6 BBB/NVU and glial modulation

BBB integrity limits the entry of peripheral inflammatory signals into the central nervous system. Evidence relevant to taVNS derives mainly from studies of vascular cognitive impairment and stroke rather than direct PND research. In a rat model of vascular cognitive impairment, taVNS was associated with reduced BBB permeability, increased hippocampal angiogenesis, attenuated astrocyte activation, and upregulated Wnt7/β-catenin signaling (91). Studies in stroke likewise suggest that taVNS may improve neurovascular-unit function (37). These findings support the biological plausibility of barrier protection but cannot be directly extrapolated to perioperative efficacy. No clinical study has yet used perioperative BBB permeability, tight-junction proteins, or neurovascular-unit measures as a primary endpoint. Whether taVNS prevents the postoperative spread of peripheral inflammation into the brain therefore remains unresolved.

Non-perioperative preclinical models suggest that taVNS can modulate microglial and astrocytic responses. In a chronic unpredictable mild-stress model, taVNS attenuated hippocampal microglial activation and reduced NF-κB p65 and IL-1β, while α7nAChR deletion weakened the effect (80). In vascular cognitive impairment, taVNS was associated with reduced astrocyte activation and enhanced Wnt7/β-catenin signaling (91). Other neurodevelopmental models have shown reductions in IL-17A signaling and pro-inflammatory microglial responses (92). These results identify glia as possible central effectors of taVNS but do not establish their role in PND. In a perioperative randomized trial, early cognitive decline defined using the original POCD construct occurred in 10.2% of the taVNS group and 32.7% of the sham group, with accompanying reductions in S100β and IL-6 (42). Because brain glial activity was not directly measured, concurrent changes in biomarkers and cognition provide only indirect support and do not establish glial remodeling as the mediator of benefit.

### 4.7 Sleep, pain, arousal, and cognitive-network modulation

Postoperative sleep disturbance, pain, and opioid exposure are all associated with PND risk. taVNS may influence arousal and sleep through the LC–NE system, but changes in pupillary responses and HRV have been observed mainly in physiological studies of healthy participants and provide only indirect evidence of target engagement (93, 94). More direct perioperative evidence comes from a study in older adults undergoing non-cardiac surgery: POD occurred in 12.0% of the taVNS group and 25.3% of controls, and mediation analysis suggested that improved sleep quality explained part of the association (44). Changes in default-mode and vigilance-network connectivity in patients with primary insomnia provide indirect network-level support for sleep modulation (71). Analgesic and opioid-sparing effects remain exploratory or secondary outcomes in most perioperative studies (5) and cannot yet be considered independent mechanisms for reducing PND risk.

taVNS may affect the prefrontal cortex, hippocampus, insula, cingulate cortex, default mode network, and other cognition-related networks, but current evidence comes mainly from healthy participants and patients with mild cognitive impairment or stroke. Different stimulation frequencies and laterality have altered limbic, insular, prefrontal, and default-mode connectivity in healthy adults (95). Studies in mild cognitive impairment have reported changes in connectivity between the hippocampus and semantic or salience networks (96), while memory-consolidation studies have shown enhanced interhemispheric and LC-related connectivity after stimulation (97). Functional near-infrared spectroscopy has linked task-related cortical hemodynamic changes to working-memory performance (98), and a post-stroke case study reported signs of prefrontal white-matter remodeling (99). Findings are not uniform across cognitive domains; negative or inconsistent results have been reported for cognitive flexibility (100). These studies support the plausibility of network plasticity but do not show that equivalent network changes mediate PND improvement in perioperative patients.

### 4.8 Preclinical and non-perioperative evidence

Preclinical studies provide indirect support for the biological plausibility of taVNS-related cognitive effects. Across animal models, auricular stimulation has been associated with changes in memory performance, functional connectivity, neuroinflammatory signaling, glial activity, and sleep-related processes (65, 97, 101, 102). However, most of these models were not designed to reproduce postoperative PND and therefore cannot establish perioperative clinical efficacy or pathway-specific mediation.

Non-perioperative human studies provide additional but indirect evidence. Studies in mild cognitive impairment, post-stroke cognitive impairment, schizophrenia, Parkinson’s disease, and healthy participants have reported changes in cognitive performance, memory, arousal, hippocampal or prefrontal connectivity, electrophysiological or multimodal neurophysiological measures, and white-matter-related signals (96, 99, 103–109). These findings are heterogeneous across populations and cognitive domains and should be used primarily to support biological plausibility, target-engagement hypotheses, and trial design rather than as evidence of perioperative cognitive benefit.

### 4.9 Stimulation parameters and dose–response considerations

Existing taVNS studies vary substantially in stimulation site, laterality, frequency, pulse width, current intensity, duty cycle, session duration, and total treatment exposure (79, 90, 104, 110–132). Experimental and non-perioperative studies suggest that neural and physiological responses may depend on stimulation parameters, but these parameters frequently vary simultaneously, preventing attribution of clinical effects to any single frequency, intensity, or waveform. Current evidence therefore does not support a universal optimal dose (113, 127).

Timing is also likely to be relevant in perioperative applications. Preoperative stimulation may serve as a preconditioning strategy, intraoperative stimulation may overlap with the period of surgical stress, and early postoperative stimulation may target the inflammatory-resolution phase. However, no standardized perioperative regimen has been established, and comparative evidence is insufficient to define an optimal stimulation window (5, 127, 131, 133).

taVNS avoids the implantation risks associated with cervical VNS and is generally associated with mild local sensory or skin reactions, while occasional dizziness or headache has also been reported (34, 36, 90, 104, 132). Nevertheless, current perioperative samples remain too small to establish the frequency of uncommon cardiovascular or autonomic adverse events. Safety and tolerability should therefore be assessed and reported systematically alongside cognitive outcomes.

## 5 Discussion

### 5.1 Principal findings

This integrated review identified four randomized perioperative taVNS trials with postoperative cognitive or delirium outcomes. The direction of the direct clinical findings favored taVNS, but each outcome family was supported by only one or two small trials. Two studies contributed to an exploratory binary synthesis, one trial reported a separate POD signal, and one reported a more favorable serial MoCA trajectory. Certainty was low to very low, and the findings do not justify routine clinical implementation.

### 5.2 Comparison with previous reviews and added value

Compared with broader taVNS cognition reviews and mechanistic summaries, this review focuses on direct perioperative randomized evidence and keeps POD, dNCR, traditional POCD, and serial MoCA trajectories separate. Only two clinically related early cognitive-decline outcomes were pooled, and the pooled estimate was explicitly labeled exploratory. The review also combines outcome-specific RoB 2 and GRADE appraisal with comparison of stimulation dose, sham design, and blinding, while maintaining a separate narrative synthesis for mechanistic and translational evidence. Biomarker changes are treated as biological plausibility signals rather than proof of mediation.

### 5.3 Interpretation within the PND nomenclature

The pooled RR of 0.34 should not be interpreted as a treatment effect for a single standardized PND outcome. The two contributing trials used related but non-interchangeable constructs—dNCR and investigator-defined traditional POCD—and the synthesis was undertaken *post hoc* only to examine concordance in the direction and approximate magnitude of effect across early postoperative cognitive deterioration. It therefore provides hypothesis-generating quantitative context rather than a confirmatory estimate for a homogeneous clinical endpoint. POD is an acute fluctuating syndrome, postoperative NCD uses a later diagnostic window, and serial MoCA scores are screening trajectories rather than diagnostic entities. Although the reported between-group differences at one and three months favored taVNS, their clinical importance cannot be established from the available data because complete precision estimates, formal PND diagnostic criteria, and patient-centered functional outcomes were not reported. Statistical differences in a cognitive screening score should therefore not be interpreted as demonstrated preservation of daily functioning, independence, quality of life, or long-term cognitive status. The most defensible interpretation is a preliminary cognitive signal that requires confirmation using standardized neuropsychological definitions and patient-centered outcomes.

### 5.4 Biological plausibility and limits of mechanistic inference

The ABVN–NTS anatomical pathway, inflammatory-reflex models, the splenic sympathetic–cholinergic immune-cell axis, autonomic regulation, BBB/NVU biology, sleep and pain pathways, and cognitive-network modulation together provide a biologically coherent rationale. However, parallel changes in IL-6, S100β, HRV, sleep, or connectivity do not demonstrate mediation. None of the included perioperative trials directly demonstrated selective ABVN engagement. No included trial directly established that CAP engagement, BBB preservation, glial modulation, or network change was necessary for cognitive benefit. Mechanistic claims should therefore be stated as supported plausibility or testable hypotheses, not as established causal pathways.

### 5.5 Potential synergy with multimodal perioperative brain protection

There is almost no direct PND evidence on synergistic effects between taVNS and other perioperative interventions. In stroke rehabilitation, closed-loop taVNS synchronized with electroencephalography-triggered brain–computer-interface training has been developed (114), and respiratory-gated auricular vagal-afferent stimulation has been used to modulate NTS- and LC-related pathways (115). These findings cannot be directly extrapolated to surgical patients. Because taVNS may influence the inflammatory reflex, sleep, and pain, combinations with prehabilitation, multimodal analgesia, sleep management, or anti-inflammatory strategies are biologically plausible (35, 71, 102). Whether these combinations are additive or synergistic, and whether they increase bradycardia, skin discomfort, or implementation burden, must be tested in prospectively designed factorial trials.

### 5.6 Clinical implications

The available randomized, biomarker, physiological, and mechanistic findings support continued investigation of taVNS as a non-invasive perioperative brain-protection strategy. Its practical ability to be delivered before, during, or after surgery, together with its potential to engage inflammatory, autonomic, sleep, pain, and cognitive-network pathways, provides a rationale for larger confirmatory trials. Current evidence is insufficient for protocolized clinical implementation, but the exploratory effect-size estimates and identified methodological limitations can inform patient selection, stimulation windows, comparator design, and outcome selection in the next generation of studies.

The available evidence is also insufficient to establish the safety of perioperative taVNS. Zhou et al. reported cardiovascular and PONV events by group, but these perioperative events were not attributed to the device; Ma et al. reported four mild local reactions across both groups and no serious AEs. Shi reported how AEs were ascertained without group-specific results, and Li did not provide structured device-AE counts. The total sample remains too small to detect uncommon cardiovascular or autonomic complications. Future trials should prospectively define AEs and ascertainment, report attribution and group-specific counts, and document withdrawals due to AEs and serious events.

### 5.7 Strengths, limitations, and added value of the integrated review

This review combines a registered four-database systematic evaluation of direct perioperative randomized evidence with an extensive structured narrative synthesis centered on neuroimmune, autonomic, sleep-related, and translational mechanisms. It distinguishes POD, dNCR, traditional POCD, postoperative NCD, and longitudinal cognitive scores instead of collapsing them into a single endpoint, and it integrates RoB 2, GRADE, stimulation-parameter analysis, and a translational framework for precision neuromodulation. The principal limitations arise from the current evidence base itself: only four randomized perioperative trials were available, and only two contributed to exploratory pooling. Importantly, the two trials contributing to the pooled estimate did not test equivalent taVNS regimens or equivalent comparator conditions; differences in stimulation dose, timing, and sham design mean that the pooled RR should not be interpreted as the effect of a single clearly defined and reproducible taVNS intervention. Several mechanistic inferences also continue to rely largely on preclinical or non-perioperative data. These constraints substantially limit confidence in efficacy estimates while preserving the rationale for further investigation.

In addition, the cross-construct synthesis of dNCR and investigator-defined traditional POCD was introduced *post hoc* and was not prespecified in the PROSPERO registration. Although this deviation was undertaken only after the limited eligible evidence base became apparent and did not alter study eligibility or selection, it increases the exploratory nature of the pooled estimate and should be considered when interpreting the result.

A further limitation concerns the mechanistic and translational component, which was a structured narrative synthesis rather than an exhaustive systematic review. Although sources were organized according to predefined thematic domains and evidence directness, literature identification and classification were not performed as independent duplicate systematic screening. Yuhan Meng organized and classified the evidence library, and Ruyi Xia subsequently cross-checked source relevance and classification. Accordingly, selective retrieval and evidence-selection bias cannot be excluded, particularly for the broader preclinical and non-perioperative literature. The mechanistic synthesis should therefore be interpreted as structured support for biological plausibility and hypothesis generation rather than as an exhaustive or quantitatively unbiased representation of the mechanistic literature.

## 6 Key evidence gaps and priorities for clinical translation

### 6.1 Standardization of stimulation dose, device reporting, and sham design

A major limitation of the current evidence base is the substantial heterogeneity in stimulation target, laterality, frequency, pulse width, current intensity, duty cycle, treatment timing, session duration, and total exposure. Device reporting is also incomplete, particularly for waveform, electrode geometry, contact area, current density, and impedance. These differences limit reproducibility and prevent meaningful dose–response inference (68, 127). Future studies should use standardized reporting frameworks and should evaluate stimulation dose as a multidimensional construct rather than as frequency or current intensity alone.

Sham design remains an additional methodological challenge. Earlobe or other non-vagal auricular stimulation may not be physiologically inert, whereas zero-current controls may compromise participant blinding because of differences in sensory perception (42, 104, 116–118). Future trials should use credible sensory-matched comparators, report operator masking where feasible, and formally assess blinding success.

### 6.2 Target engagement and causal validation

No single currently available biomarker can confirm selective vagal-pathway engagement. HRV, inflammatory mediators, S100β, pupillary responses, electrophysiological measures, and neuroimaging may provide complementary physiological information, but each is influenced by multiple perioperative factors and should not be interpreted individually as proof of vagal target engagement (42, 68, 72, 88, 89, 93, 94, 134–138). Future studies should integrate autonomic, immune, and brain-network measures within synchronized multimodal protocols.

Establishing mechanism will also require more than parallel changes in biomarkers and cognition. Prespecified mediation analyses, repeated temporal measurements, and pathway-specific physiological assessments are needed to test whether changes in neural target engagement precede downstream immune, autonomic, network, and cognitive effects. Until such studies are available, the proposed mechanistic pathways should remain hypotheses rather than established mediators of clinical benefit.

### 6.3 Clinical trial design, standardized outcomes, and long-term follow-up

The direct perioperative evidence currently consists of only four small randomized trials with heterogeneous cognitive endpoints and limited follow-up. Future studies should use adequately powered multicenter designs, prospectively define a single primary outcome, and distinguish POD, dNCR, postoperative NCD, and longitudinal cognitive-screening measures rather than combining them into a single PND endpoint. POD should be assessed repeatedly using validated instruments, whereas dNCR and postoperative NCD should be evaluated using baseline-adjusted neuropsychological testing across multiple cognitive domains.

Longer-term patient-centered outcomes are also needed. Follow-up should extend beyond the immediate postoperative period and should include cognitive trajectories, functional independence, activities of daily living, quality of life, readmission, care needs, and adverse events. Statistical analysis plans should prespecify the handling of repeated measurements, attrition, and missing outcomes. These features will be necessary to determine whether short-term cognitive signals translate into clinically meaningful and durable benefit.

### 6.4 Risk-enriched and adaptive development strategies

Future trials may improve efficiency by prospectively identifying patients at greater risk of PND, including those with advanced age, frailty, baseline cognitive impairment, autonomic dysfunction, or increased inflammatory burden. Such characteristics should be used as prespecified stratification variables rather than as *post hoc* definitions of treatment response.

Adaptive or closed-loop stimulation approaches may ultimately allow taVNS dose to be individualized according to physiological responses, but this concept remains investigational. HRV, pupillary responses, electroencephalographic measures, sleep state, or inflammatory markers could potentially contribute to feedback-guided stimulation, provided that their validity as target-engagement measures is established first. For the present evidence base, priority should remain on reproducible stimulation protocols, credible controls, standardized outcomes, and adequately powered confirmatory randomized trials.

## 7 Conclusion

taVNS has a biologically plausible neuroanatomical and neuroimmune rationale for investigation as a perioperative neuromodulatory strategy. Auricular stimulation is intended to engage vagal afferents and may influence NTS-related brainstem networks, including pathways involving the locus coeruleus, hypothalamus, limbic system, prefrontal regions, and peripheral neuroimmune regulation. Proposed downstream mechanisms include modulation of inflammatory-reflex signaling, the splenic sympathetic–cholinergic immune-cell axis, autonomic regulation, BBB/NVU function, sleep and pain, and cognitive-network activity (34, 35, 37, 67, 70). However, none of the included perioperative trials directly demonstrated selective ABVN engagement or established any of these pathways as mediators of cognitive benefit. Four small randomized perioperative trials reported findings in a favorable direction across distinct early cognitive, delirium, and longitudinal screening outcomes, with accompanying biomarker and sleep-related changes that are consistent with biological plausibility but do not establish pathway-specific target engagement or mediation (42–45). However, certainty is low to very low, and efficacy remains unconfirmed. Because stimulation protocols and comparator conditions differed materially across trials, the available clinical signal cannot be attributed to a single standardized or reproducible taVNS regimen. Standardized PND phenotypes, reproducible stimulation-dose reporting, credible sensory-matched controls, multimodal target-engagement measures, risk-enriched trial populations, and long-term patient-centered outcomes will be central to defining the optimal therapeutic window. Current evidence supports confirmatory research rather than routine clinical practice.

## Data Availability

Study-level data underlying the exploratory quantitative synthesis are provided in Supplementary Data S6, and the analysis code is provided in Supplementary Code S7. The Supplementary materials include Supplementary Methods S1, S1.5; Supplementary Tables S2–S5a,S5b; Supplementary Data S6; Supplementary Code S7; and Supplementary Table S8. A separate Supplementary File S9 contains the original date-stamped PROSPERO registration record (CRD420261468039; Version 1.0, published 4 August 2026). No individual-participant data were obtained or analyzed.

## Glossary

ABVN: auricular branch of the vagus nerve
BBB: blood–brain barrier
BDNF: brain-derived neurotrophic factor
CAM: Confusion Assessment Method
CAM-ICU: Confusion Assessment Method for the Intensive Care Unit
CAP: cholinergic anti-inflammatory pathway
ChAT: choline acetyltransferase
CI: confidence interval
DAMPs: damage-associated molecular patterns
dNCR: delayed neurocognitive recovery
fMRI: functional magnetic resonance imaging
GRADE: Grading of Recommendations Assessment Development and Evaluation
HRV: heart rate variability
HPA: hypothalamic–pituitary–adrenal
IL: interleukin
LC: locus coeruleus
LC–NE: locus-coeruleus–noradrenergic
MD: mean difference
MMSE: Mini-Mental State Examination
MoCA: Montreal Cognitive Assessment
NCD: neurocognitive disorder
NF-κB: nuclear factor kappa B
NLRP3: NOD-like receptor family pyrin domain-containing 3
NTS: nucleus tractus solitarius
PND: perioperative neurocognitive disorders
POD: postoperative delirium
POCD: postoperative cognitive dysfunction
PRISMA: Preferred Reporting Items for Systematic Reviews and Meta-Analyses
RCT: randomized controlled trial
RR: risk ratio
RoB 2: Cochrane Risk of Bias 2 tool
taVNS: transcutaneous auricular vagus nerve stimulation
TNF-α: tumor necrosis factor alpha
VNS: vagus nerve stimulation.
