# Supplementary material for: Transcutaneous auricular vagus nerve stimulation in perioperative neurocognitive disorders: clinical evidence and mechanistic perspectives
Source: Front Neurol. 2026 Oct 29;17:1963894. doi: 10.3389/fneur.2026.1963894 (PMC13754654; doi:10.3389/fneur.2026.1963894)
Supplement: Supplementary file 1 [file Data_Sheet_1.pdf]

NIHR | National Institute for Health and Care Research

PROSPERO
International prospective register of systematic reviews

Effects of transcutaneous auricular vagus nerve stimulation on perioperative neurocognitive outcomes in adults undergoing surgery: a systematic review and meta-analysis

Ruyi Xia, Yuhan Meng

REVIEW TITLE AND BASIC DETAILS

Review title

Effects of transcutaneous auricular vagus nerve stimulation on perioperative neurocognitive outcomes in adults undergoing surgery: a systematic review and meta-analysis

Condition or domain being studied

Postoperative delirium; Cognitive impairment

Perioperative neurocognitive disorders and postoperative cognitive outcomes, including postoperative delirium, delayed neurocognitive recovery, postoperative neurocognitive disorder, investigator-defined postoperative cognitive dysfunction, and postoperative changes in validated cognitive scores. These outcome constructs and time windows will be synthesized separately rather than combined as a single PND incidence outcome.

Rationale for the review

Perioperative neurocognitive disorders are clinically important complications associated with impaired recovery and longer-term functional consequences. Transcutaneous auricular vagus nerve stimulation is a non-invasive intervention that may influence autonomic, inflammatory, sleep, pain and cognitive pathways, but perioperative studies use heterogeneous stimulation protocols, comparators, cognitive constructs and assessment windows. Postoperative delirium, consensus-defined delayed neurocognitive recovery, investigator-defined historical postoperative cognitive dysfunction and changes in cognitive scale scores are not interchangeable outcomes. This review will systematically evaluate the efficacy and safety of perioperative taVNS in adults undergoing surgery, distinguish randomized from quasi-randomized evidence, separate clinically different outcome constructs and time windows, and identify when quantitative pooling is justified. It will also clarify uncertainties related to sham design, delivered dose, risk of bias, safety reporting and the applicability of current evidence to perioperative practice.

Review objectives
In adults undergoing surgery, does perioperative transcutaneous auricular vagus nerve stimulation, compared with sham stimulation, non-vagal auricular stimulation, no stimulation, usual care, or another eligible control, reduce postoperative delirium or early postoperative cognitive decline, improve postoperative cognitive outcomes, and remain acceptably safe? The review will analyze postoperative delirium, consensus-defined delayed neurocognitive recovery, investigator-defined historical postoperative cognitive dysfunction, study-defined early cognitive decline, continuous cognitive scores, long-term postoperative neurocognitive disorder, and safety as distinct outcomes. It will also examine whether effects differ according to study design, surgical population, stimulation timing and dose, stimulation site and side, comparator credibility, assessment window, and risk of bias, where the available number of studies permits.

Keywords
Transcutaneous auricular vagus nerve stimulation; Postoperative cognitive dysfunction; Perioperative care

Country
China

ELIGIBILITY CRITERIA

Population

Included
Adults aged 18 years or older undergoing elective or emergency surgery under general, regional, neuraxial, or combined anesthesia. Studies from any surgical specialty will be eligible, including studies enrolling only older adults and studies with mixed adult populations. Eligible taVNS may be delivered during the preoperative, intraoperative, or postoperative period.

Excluded
Children or adolescents; healthy volunteers without surgery; non-surgical populations; animal or in-vitro studies; patients receiving taVNS only for chronic neurological, psychiatric, pain, or rehabilitation disorders unrelated to surgery; and studies without a postoperative cognitive, delirium, neuropsychological, or prespecified safety outcome.

Intervention(s) or exposure(s)

Included
Transcutaneous auricular vagus nerve stimulation, transauricular vagus nerve stimulation, auricular vagus nerve stimulation, taVNS or ta-VNS, or another non-invasive electrical stimulation explicitly intended to recruit the auricular branch of the vagus nerve. Stimulation may be delivered before, during or after surgery. Ear region, side, frequency, pulse width, waveform, intensity, current-adjustment method, duty cycle, session duration, number of sessions, perioperative timing, device, electrode, impedance and adherence will be extracted.

Excluded

Implanted cervical vagus nerve stimulation; transcutaneous cervical vagus nerve stimulation; auricular acupuncture without electrical stimulation; ear stimulation without a stated or reasonably identifiable vagal target; and multicomponent interventions in which the independent effect of taVNS cannot be determined.

Comparator(s) or control(s)

Included
Eligible comparators are earlobe sham, active non-vagal auricular stimulation, identical electrode placement with 0 mA or transient stimulation, no stimulation, usual perioperative care, or another active comparator from which the taVNS effect can be isolated. Comparators will be coded separately as active sensory-matched sham, inactive or 0-mA sham, blank or no-treatment control, usual care, or other active comparator and will not be assumed equivalent.

Study design

Both randomized and nonrandomized study types will be included.

Included
Randomized controlled trials, including cluster-randomized trials where applicable, will be included. Crossover randomized trials will be eligible only when perioperative data can be extracted without unacceptable carryover. Quasi-randomized controlled studies may contribute to an expanded qualitative synthesis and sensitivity analyses but will not be combined by default with truly randomized trials. The primary efficacy meta-analysis will prioritize randomized controlled trials.

Excluded
Single-arm studies, case reports, case series, uncontrolled observational studies, reviews, editorials and commentaries will be excluded. Protocol-only or registry-only records without results will not enter efficacy synthesis but may be described as ongoing evidence.

Context

Inpatient or day-case perioperative care in any surgical specialty and any hospital setting. Eligible stimulation may occur before anesthesia or surgery, intraoperatively, or during postoperative recovery. Studies must evaluate postoperative cognitive, delirium, neuropsychological, or prespecified safety outcomes.

TIMELINE OF THE REVIEW

Date of first submission to PROSPERO

04 August 2026

Review timeline

Review start date: 1 August 2026. Review end date: 31 March 2027.

Date of registration in PROSPERO

04 August 2026

AVAILABILITY OF FULL PROTOCOL

Availability of full protocol
A full protocol has not been written.

SEARCHING AND SCREENING

Search for unpublished studies
Both published and unpublished studies will be sought.

Main sources that will be searched
The main sources to be searched are *CENTRAL - Cochrane Central Register of Controlled Trials*, *Embase* and *PubMed*.

Other sources that will be searched
Web of Science Core Collection

Search language restrictions
There are no language restrictions.

Search date restrictions
There are no search date restrictions.

Other methods of identifying studies
Other studies will be identified by: *looking through all the articles that cite the papers included in the review ("snowballing" or forward citation searching)*, *reference list checking (backward citation searching)* and *searching trial or study registers*.

Additional information about identifying studies
Reference lists of included studies and relevant reviews will be screened, and forward citation searching will be performed. Trial registries will be searched for ongoing, completed, and unpublished studies. Study authors will be contacted when essential methodological or numerical information is unavailable.

Link to search strategy
A full search strategy has been uploaded to PROSPERO. The PDF may be accessed through this link
https://www.crd.york.ac.uk/PROSPEROFILES/0b5ec932922449599333e45fbd1a598b.pdf.

Selection process
Studies will be screened independently by at least two people (or person/machine combination) with a process to resolve differences.

Other relevant information about searching and screening
Search results will be imported into reference-management software and duplicates will be removed before screening. Two reviewers will independently screen titles and abstracts and subsequently assess potentially eligible full texts. Disagreements will be resolved through discussion or adjudication by a third reviewer. Reasons for full-text exclusion will be recorded. Searches will be updated before the final analysis.

DATA COLLECTION PROCESS

Data extraction from published articles and reports
Data will be extracted independently by at least two people (or person/machine combination) with a process to resolve differences.

Authors will not be contacted for further information.

Study risk of bias or quality assessment
Risk of bias will be assessed using: *Cochrane RoB-2* and *ROBINS-I*

Data will be assessed independently by at least two people (or person/machine combination) with a process to resolve differences.

Additional information will be sought from study investigators if required information is unclear or unavailable in the study publications/reports.

Reporting bias assessment
Trial registries, protocols, statistical analysis plans and final reports will be compared for omitted outcomes or time points. Unpublished studies will be sought and authors contacted. Funnel plots and Egger's test will be used only when at least 10 comparable studies are available; otherwise reporting bias will be assessed qualitatively.

Certainty assessment
The certainty of evidence for each critical outcome will be assessed using the GRADE approach. Randomized trials will initially be rated as high-certainty evidence, while non-randomized or quasi-randomized studies will initially be rated as low certainty. Certainty may be downgraded for risk of bias, inconsistency, indirectness, imprecision, and publication bias. Where appropriate, certainty may be upgraded according to established GRADE criteria. Two reviewers will independently assess certainty, with disagreements resolved by consensus or a third reviewer. Evidence will be rated as high, moderate, low, or very low, and summarized in outcome-specific Summary of Findings tables.

OUTCOMES TO BE ANALYSED

Main outcomes
Two co-primary outcomes will be analyzed separately. (1) Postoperative delirium incidence, diagnosed using CAM, 3D-CAM, CAM-ICU, DSM criteria, or another validated tool, primarily during postoperative days 0–7 or before discharge; assessment frequency, observation days, duration and severity will also be recorded. (2) Early postoperative cognitive decline within 30 days, stratified as consensus-defined delayed neurocognitive recovery, investigator-defined historical postoperative cognitive dysfunction, or other study-defined binary early cognitive decline. These constructs will not be pooled unconditionally. Binary outcomes will be summarized as risk ratios with 95% confidence intervals; risk differences may be supplementary. Outcomes from materially different time windows will be analyzed separately.

Additional outcomes
Additional outcomes are continuous MoCA and MMSE scores; composite and domain-specific neuropsychological outcomes; postoperative neurocognitive disorder from 30 days to 12 months; and cognitive outcomes at 0–3, 4–7, 8–30, 31–90, and more than 90 days. Mechanistic and recovery outcomes include IL-6, TNF-alpha, IL-1beta, S100beta, NSE, CRP,

neutrophil-to-lymphocyte ratio, heart-rate variability and other autonomic measures, sleep, pain and opioid consumption, length of stay, functional recovery, and quality of life. Safety outcomes include all adverse events, bradycardia, arrhythmia, local skin or ear reactions, stimulation interruption, withdrawal due to adverse events, serious adverse events, adherence, and completed sessions. Biomarkers will not be combined with clinical cognitive outcomes. Continuous outcomes will use mean difference when the same scale is used and standardized mean difference only for the same construct measured with different scales; change scores and follow-up values will not be mixed without a justified method.

PLANNED DATA SYNTHESIS

Strategy for data synthesis

Clinical and methodological comparability will be assessed before pooling. Postoperative delirium, consensus-defined delayed neurocognitive recovery, investigator-defined historical postoperative cognitive dysfunction, isolated MoCA or MMSE scores, and long-term postoperative neurocognitive disorder will be analyzed separately. Outcomes from substantially different postoperative time windows will not be pooled. Quantitative pooling will be undertaken only when at least two studies are sufficiently comparable in population, intervention, comparator, outcome construct, and assessment window; analyses based on only two studies will be labelled exploratory. Random-effects meta-analysis will be used when appropriate, with the between-study variance estimator finalized in the prespecified statistical analysis plan; fixed-effect models will be sensitivity analyses. Q, I-squared, and tau-squared will be reported, and I-squared of zero with very few studies will not be interpreted as proof of clinical homogeneity. Randomized trials will form the primary efficacy analysis; quasi-randomized studies will be analyzed separately or in sensitivity analyses. Multi-arm studies will use the most credible sensory-matched comparator for the main analysis, with shared groups split or eligible controls combined to avoid double counting. Planned subgroup and sensitivity analyses will be conducted only when study numbers permit. Funnel plots and small-study-effect tests will be considered only when at least 10 independent studies are available for the same outcome; otherwise publication bias will be discussed qualitatively. Certainty of evidence will be assessed using GRADE for each clinically distinct outcome. If pooling is not justified, structured narrative synthesis and study-level effect estimates will be presented.

CURRENT REVIEW STAGE

Stage of the review at this submission

Review stage	Started	Completed
Pilot work		
Formal searching/study identification		
Screening search results against inclusion criteria		
Data extraction or receipt of IPD		
Risk of bias/quality assessment		

Review stage	Started	Completed
Data synthesis		

Review status
The review is currently planned or ongoing.

Publication of review results
Results of the review will be published in English.

REVIEW AFFILIATION, FUNDING AND PEER REVIEW

Review team members
Dr Ruyi Xia (review guarantor and contact) ORCID: 0009-0007-2162-3521. Yantai yuhuangding hospital. China.

No conflict of interest declared.

Dr Yuhan Meng. Yantai yuhuangding hospital. China.

No conflict of interest declared.

Named contact
Dr Ruyi Xia (15854862723@163.com). ORCID: 0009-0007-2162-3521. Yantai yuhuangding hospital. China.

Review affiliation
Yantai Yuhuangding Hospital

Funding source

Grant number
ZD202504

Additional non-commercial funding information
Scientific Research Foundation of Yantai Yuhuangding Hospital

Additional information about funding
This review is supported by the Scientific Research Foundation of Yantai Yuhuangding Hospital (Grant No. ZD202504), entitled "Metabolic regulatory network and biomarkers of perioperative neurocognitive disorders."

Peer review
There has been no peer review of this planned review.

ADDITIONAL INFORMATION

Review conflict of interest
Declared individual interests are recorded under team member details.. No additional interests are recorded for this review.

Medical Subject Headings
Vagus Nerve Stimulation; Transcutaneous Electric Nerve Stimulation; Postoperative Cognitive Complications; Delirium; Neurocognitive Disorders; Surgical Procedures,

Operative; Aged

SIMILAR REVIEWS

Check for similar records already in PROSPERO

PROSPERO identified a number of existing PROSPERO records that were similar to this one (last check made on 3 August 2026). These are shown below along with the reasons given by that the review team for the reviews being different and/or proceeding.

- Transcutaneous auricular vagus nerve stimulation for postoperative gastrointestinal recovery after abdominal or pelvic surgery: a systematic review and meta-analysis of randomized controlled trials [published 19 July 2026] [CRD420261455175]. The review was judged **not to be similar**
- Perioperative Transcutaneous Auricular Vagus Nerve Stimulation for Postoperative Pain and Recovery Trajectories: A Systematic Review and Meta-Analysis of Randomized Controlled Trials [published 29 May 2026] [CRD420261406574]. The review was judged **not to be similar**
- Effects of electrical vagus nerve stimulation on postoperative quality of recovery and gastrointestinal outcomes in surgical patients: protocol for a systematic review and meta-analysis of randomized controlled trials [published 2 June 2026] [CRD420261409898]. The review was judged **not to be similar**

PROSPERO version history

- Version 1.0, published 04 Aug 2026

Disclaimer

The content of this record displays the information provided by the review team. PROSPERO does not peer review registration records or endorse their content.

PROSPERO accepts and posts the information provided in good faith; responsibility for record content rests with the review team. The guarantor for this record has affirmed that the information provided is truthful and that they understand that deliberate provision of inaccurate information may be construed as scientific misconduct.

PROSPERO does not accept any liability for the content provided in this record or for its use. Readers use the information provided in this record at their own risk.

Any enquiries about the record should be referred to the named review contact
