# Supplementary material for: Transcutaneous auricular vagus nerve stimulation in perioperative neurocognitive disorders: clinical evidence and mechanistic perspectives
Source: Front Neurol. 2026 Oct 29;17:1963894. doi: 10.3389/fneur.2026.1963894 (PMC13754654; doi:10.3389/fneur.2026.1963894)
Supplement: Supplementary file 2 [file Supplementary_file_1.docx]

**Supplementary Materials**

Full search strategies, protocol and registration records, PRISMA 2020 checklist, detailed RoB 2 judgments, GRADE evidence profiles, study-level meta-analysis data, analysis code, and study-level adverse-event reporting.

**Contents**

• Supplementary Methods S1. Full electronic search strategies

• Supplementary Methods S1.5. Identification and classification of mechanistic and translational evidence

• Supplementary Table S2. Protocols and trial registrations identified during screening

• Supplementary Table S3. PRISMA 2020 reporting checklist and locations

• Supplementary Table S4. Detailed Cochrane RoB 2 judgments and supporting rationale

• Supplementary Tables S5a-S5b. GRADE evidence profiles and summary of findings

• Supplementary Data S6. Study-level data used for the exploratory quantitative synthesis

• Supplementary Code S7. Python code used for the exploratory quantitative synthesis

• Supplementary Table S8. Study-level adverse-event reporting in included perioperative randomized trials

• Supplementary File S9. Original date-stamped PROSPERO registration record (CRD420261468039; Version 1.0, published 4 August 2026)

*The locations in the PRISMA checklist refer to the embedded systematic-review component. Reported database yields are those recorded in the review search log. Search syntax is presented in database-specific reproducible form corresponding to the final high-sensitivity concept structure.*

# Supplementary Methods S1. Full electronic search strategies

The searches covered each database from inception through 5 August 2026. No language, publication-year, or document-type restrictions were applied at the search stage. Trial registries, reference lists, and forward citations were checked separately.

| **Database** | **Platform** | **Search date** | **Coverage / restrictions** | **Records retrieved** |
| --- | --- | --- | --- | --- |
| PubMed/MEDLINE | NCBI PubMed | 5 August 2026 | Inception to search date; no language, year, or publication-type restriction | 153 |
| Embase | Embase.com | 5 August 2026 | Inception to search date; no language, year, or publication-type restriction | 450 |
| Web of Science Core Collection | Web of Science | 5 August 2026 | All editions and all years; no language or document-type restriction | 126 |
| CENTRAL | Cochrane Library | 5 August 2026 | Inception to search date; Trials; no language or year restriction | 249 |

*Total database records before deduplication: 978.*

## S1.1 PubMed/MEDLINE

Platform: NCBI PubMed; search date: 5 August 2026; records retrieved: n = 153.

(
 "Vagus Nerve Stimulation"[Mesh]
 OR "Transcutaneous Electric Nerve Stimulation"[Mesh]
 OR "transcutaneous auricular vagus nerve stimulation"[tiab]
 OR "transauricular vagus nerve stimulation"[tiab]
 OR "auricular vagus nerve stimulation"[tiab]
 OR "auricular vagal stimulation"[tiab]
 OR "auricular branch vagus nerve stimulation"[tiab]
 OR "transcutaneous vagus nerve stimulation"[tiab]
 OR "noninvasive vagus nerve stimulation"[tiab]
 OR "non-invasive vagus nerve stimulation"[tiab]
 OR "low-level tragus stimulation"[tiab]
 OR "low level tragus stimulation"[tiab]
 OR "tragus stimulation"[tiab]
 OR "ear vagus stimulation"[tiab]
 OR taVNS[tiab]
 OR tVNS[tiab]
 OR aVNS[tiab]
 OR (
 (auricular[tiab] OR transauricular[tiab] OR "external ear"[tiab] OR tragus[tiab] OR concha[tiab] OR cymba[tiab])
 AND (vagus[tiab] OR vagal[tiab])
 AND (stimulat*[tiab] OR neuromodulat*[tiab])
 )
)
AND
(
 "Surgical Procedures, Operative"[Mesh]
 OR "Perioperative Care"[Mesh]
 OR "Postoperative Period"[Mesh]
 OR "Anesthesia"[Mesh]
 OR surger*[tiab]
 OR surgical[tiab]
 OR operation*[tiab]
 OR operative[tiab]
 OR perioperativ*[tiab]
 OR peri-operativ*[tiab]
 OR postoperativ*[tiab]
 OR post-operativ*[tiab]
 OR anesthesia[tiab]
 OR anaesthesia[tiab]
 OR anesthetic*[tiab]
 OR anaesthetic*[tiab]
)
AND
(
 "Delirium"[Mesh]
 OR "Cognition Disorders"[Mesh]
 OR "Neurocognitive Disorders"[Mesh]
 OR "Neuropsychological Tests"[Mesh]
 OR delirium[tiab]
 OR cognit*[tiab]
 OR neurocognit*[tiab]
 OR "postoperative delirium"[tiab]
 OR "perioperative neurocognitive disorder"[tiab]
 OR "perioperative neurocognitive disorders"[tiab]
 OR "postoperative neurocognitive disorder"[tiab]
 OR "postoperative neurocognitive disorders"[tiab]
 OR "postoperative cognitive dysfunction"[tiab]
 OR "postoperative cognitive impairment"[tiab]
 OR "early postoperative cognitive decline"[tiab]
 OR "delayed neurocognitive recovery"[tiab]
 OR POCD[tiab]
 OR dNCR[tiab]
 OR MoCA[tiab]
 OR MMSE[tiab]
 OR "Montreal Cognitive Assessment"[tiab]
 OR "Mini Mental State Examination"[tiab]
 OR neuropsycholog*[tiab]
 OR memory[tiab]
 OR attention[tiab]
 OR "executive function"[tiab]
 OR CAM[tiab]
 OR CAM-ICU[tiab]
)

## S1.2 Embase

Platform: Embase.com; search date: 5 August 2026; records retrieved: n = 450.

(
 'vagus nerve stimulation'/exp
 OR 'transcutaneous electrical nerve stimulation'/exp
 OR 'transcutaneous auricular vagus nerve stimulation':ti,ab,kw
 OR 'transauricular vagus nerve stimulation':ti,ab,kw
 OR 'auricular vagus nerve stimulation':ti,ab,kw
 OR 'auricular vagal stimulation':ti,ab,kw
 OR 'auricular branch vagus nerve stimulation':ti,ab,kw
 OR 'transcutaneous vagus nerve stimulation':ti,ab,kw
 OR 'noninvasive vagus nerve stimulation':ti,ab,kw
 OR 'non-invasive vagus nerve stimulation':ti,ab,kw
 OR 'low-level tragus stimulation':ti,ab,kw
 OR 'low level tragus stimulation':ti,ab,kw
 OR 'tragus stimulation':ti,ab,kw
 OR 'ear vagus stimulation':ti,ab,kw
 OR tavns:ti,ab,kw
 OR tvns:ti,ab,kw
 OR avns:ti,ab,kw
 OR (((auricular OR transauricular OR 'external ear' OR tragus OR concha OR cymba) NEAR/3 (vagus OR vagal)) NEAR/3 (stimulat* OR neuromodulat*)):ti,ab,kw
)
AND
(
 'surgery'/exp
 OR 'surgical procedure'/exp
 OR 'perioperative period'/exp
 OR 'postoperative period'/exp
 OR 'anesthesia'/exp
 OR surger*:ti,ab,kw
 OR surgical:ti,ab,kw
 OR operation*:ti,ab,kw
 OR operative:ti,ab,kw
 OR perioperativ*:ti,ab,kw
 OR 'peri operativ*':ti,ab,kw
 OR postoperativ*:ti,ab,kw
 OR 'post operativ*':ti,ab,kw
 OR anesthesia:ti,ab,kw
 OR anaesthesia:ti,ab,kw
 OR anesthetic*:ti,ab,kw
 OR anaesthetic*:ti,ab,kw
)
AND
(
 'delirium'/exp
 OR 'cognitive defect'/exp
 OR 'neurocognitive disorder'/exp
 OR 'neuropsychological test'/exp
 OR delirium:ti,ab,kw
 OR cognit*:ti,ab,kw
 OR neurocognit*:ti,ab,kw
 OR 'postoperative delirium':ti,ab,kw
 OR 'perioperative neurocognitive disorder*':ti,ab,kw
 OR 'postoperative neurocognitive disorder*':ti,ab,kw
 OR 'postoperative cognitive dysfunction':ti,ab,kw
 OR 'postoperative cognitive impairment':ti,ab,kw
 OR 'early postoperative cognitive decline':ti,ab,kw
 OR 'delayed neurocognitive recovery':ti,ab,kw
 OR pocd:ti,ab,kw
 OR dncr:ti,ab,kw
 OR moca:ti,ab,kw
 OR mmse:ti,ab,kw
 OR 'montreal cognitive assessment':ti,ab,kw
 OR 'mini mental state examination':ti,ab,kw
 OR neuropsycholog*:ti,ab,kw
 OR memory:ti,ab,kw
 OR attention:ti,ab,kw
 OR 'executive function':ti,ab,kw
 OR cam:ti,ab,kw
 OR 'cam-icu':ti,ab,kw
)

## S1.3 Web of Science Core Collection

Platform: Web of Science; all editions; search date: 5 August 2026; records retrieved: n = 126.

TS=(
 "transcutaneous auricular vagus nerve stimulation"
 OR "transauricular vagus nerve stimulation"
 OR "auricular vagus nerve stimulation"
 OR "auricular vagal stimulation"
 OR "auricular branch vagus nerve stimulation"
 OR "transcutaneous vagus nerve stimulation"
 OR "noninvasive vagus nerve stimulation"
 OR "non-invasive vagus nerve stimulation"
 OR "low-level tragus stimulation"
 OR "low level tragus stimulation"
 OR "tragus stimulation"
 OR "ear vagus stimulation"
 OR taVNS
 OR tVNS
 OR aVNS
 OR (((auricular OR transauricular OR "external ear" OR tragus OR concha OR cymba) NEAR/3 (vagus OR vagal)) NEAR/3 (stimulat* OR neuromodulat*))
)
AND TS=(
 surgery OR surgical OR operation* OR operative
 OR perioperativ* OR peri-operativ*
 OR postoperativ* OR post-operativ*
 OR anesthesia OR anaesthesia OR anesthetic* OR anaesthetic*
 OR "surgical procedure*" OR "perioperative care" OR "postoperative period"
)
AND TS=(
 delirium OR cognition OR cognitive OR neurocognitive OR cognit* OR neurocognit*
 OR "postoperative delirium"
 OR "perioperative neurocognitive disorder*"
 OR "postoperative neurocognitive disorder*"
 OR "postoperative cognitive dysfunction"
 OR "postoperative cognitive impairment"
 OR "early postoperative cognitive decline"
 OR "delayed neurocognitive recovery"
 OR POCD OR dNCR OR MoCA OR MMSE
 OR "Montreal Cognitive Assessment"
 OR "Mini Mental State Examination"
 OR neuropsycholog* OR memory OR attention OR "executive function"
 OR CAM OR CAM-ICU
)

## S1.4 Cochrane Central Register of Controlled Trials (CENTRAL)

Platform: Cochrane Library; search date: 5 August 2026; records retrieved: n = 249.

#1 MeSH descriptor: [Vagus Nerve Stimulation] explode all trees
#2 MeSH descriptor: [Transcutaneous Electric Nerve Stimulation] explode all trees
#3 ("transcutaneous auricular vagus nerve stimulation" OR "transauricular vagus nerve stimulation" OR "auricular vagus nerve stimulation" OR "auricular vagal stimulation" OR "auricular branch vagus nerve stimulation" OR "transcutaneous vagus nerve stimulation" OR "noninvasive vagus nerve stimulation" OR "non-invasive vagus nerve stimulation" OR "low-level tragus stimulation" OR "low level tragus stimulation" OR "tragus stimulation" OR "ear vagus stimulation" OR taVNS OR tVNS OR aVNS):ti,ab,kw
#4 (((auricular OR transauricular OR "external ear" OR tragus OR concha OR cymba) NEAR/3 (vagus OR vagal)) NEAR/3 (stimulat* OR neuromodulat*)):ti,ab,kw
#5 #1 OR #2 OR #3 OR #4

#6 MeSH descriptor: [Surgical Procedures, Operative] explode all trees
#7 MeSH descriptor: [Perioperative Care] explode all trees
#8 MeSH descriptor: [Postoperative Period] explode all trees
#9 MeSH descriptor: [Anesthesia] explode all trees
#10 (surger* OR surgical OR operation* OR operative OR perioperativ* OR peri-operativ* OR postoperativ* OR post-operativ* OR anesthesia OR anaesthesia OR anesthetic* OR anaesthetic* OR "surgical procedure*" OR "perioperative care" OR "postoperative period"):ti,ab,kw
#11 #6 OR #7 OR #8 OR #9 OR #10

#12 MeSH descriptor: [Delirium] explode all trees
#13 MeSH descriptor: [Cognition Disorders] explode all trees
#14 MeSH descriptor: [Neurocognitive Disorders] explode all trees
#15 MeSH descriptor: [Neuropsychological Tests] explode all trees
#16 (delirium OR cognit* OR neurocognit* OR "postoperative delirium" OR "perioperative neurocognitive disorder*" OR "postoperative neurocognitive disorder*" OR "postoperative cognitive dysfunction" OR "postoperative cognitive impairment" OR "early postoperative cognitive decline" OR "delayed neurocognitive recovery" OR POCD OR dNCR OR MoCA OR MMSE OR "Montreal Cognitive Assessment" OR "Mini Mental State Examination" OR neuropsycholog* OR memory OR attention OR "executive function" OR CAM OR CAM-ICU):ti,ab,kw
#17 #12 OR #13 OR #14 OR #15 OR #16

#18 #5 AND #11 AND #17 in Trials

*Additional searching and registry cross-checking: ClinicalTrials.gov, the Chinese Clinical Trial Registry (ChiCTR), and the International Traditional Medicine Clinical Trial Registry (ITMCTR) were checked through 5 August 2026 using taVNS and auricular-vagus terms combined with perioperative and cognitive or delirium concepts. Reference lists and forward citations of eligible reports were also examined. Registry entries were linked at the study level to protocol or efficacy publications to avoid double counting. Registry records were used to characterize protocols and ongoing studies and were not counted as completed efficacy trials.*

## Supplementary Methods S1.5. Identification and classification of mechanistic and translational evidence

Mechanistic and translational evidence was identified separately from the systematic clinical-efficacy review through records retrieved during the clinical search, key reviews in taVNS and vagus-nerve physiology, backward and forward citation tracing, and topic-specific searches updated through 5 August 2026. The topic-specific mechanistic searches were not prospectively logged in sufficient detail to permit full reconstruction of every platform-specific search string.

Sources were retained for detailed narrative discussion when they informed at least one of the following domains: anatomical or physiological plausibility of auricular vagal stimulation; perioperative or surgery-related biological mechanisms; neural or physiological target engagement; stimulation dose, timing, or parameter selection; comparator and sham design; safety and tolerability; or translational implications for future perioperative trials.

Evidence was classified according to its directness to the perioperative review question into four categories: perioperative human evidence, surgery-related preclinical evidence, non-perioperative patient evidence, and healthy-participant target-engagement evidence. Perioperative human evidence was considered the most directly applicable to the clinical question. Preclinical and non-perioperative evidence was used to support biological plausibility, target engagement, dose selection, or hypothesis generation and was not treated as direct evidence of perioperative cognitive efficacy or causal mediation.

Original studies were prioritized for specific mechanistic claims, whereas systematic and narrative reviews were used primarily for context and citation tracing. The narrative evidence library comprised 132 individually verified sources. Yuhan Meng organized the broader evidence library and classified sources according to relevance and directness to the review question, and Ruyi Xia subsequently cross-checked source relevance and evidence classification. This structured narrative search was intended to provide coverage of the major mechanistic and translational domains rather than exhaustive systematic retrieval of every mechanistic publication.

**Supplementary Table S2. Protocols and trial registrations identified during screening**

| **Registry or protocol citation** | **Registration number** | **Population** | **Intervention** | **Planned outcome** | **Publication status** | **Linked full publication** | **Included in efficacy review** | **Reason for non-inclusion** |
| --- | --- | --- | --- | --- | --- | --- | --- | --- |
| Wu et al. VNSTAR protocol | NCT06421077 | Older adults undergoing elective surgery | taVNS vs active low-frequency control | POD and 90-day cognition/function | Protocol only | Wu et al. protocol [4] | No | No efficacy results in protocol |
| Liao et al. total knee arthroplasty protocol | Not reported in protocol publication | Adults 65–80 undergoing TKA | taVNS vs earlobe/antihelix sham | POD and longer-term cognitive outcomes | Protocol only | Liao et al. protocol [5] | No | No efficacy results in protocol |
| Zhou et al. hip-fracture protocol | ChiCTR2400085508 | Older adults undergoing hip-fracture surgery | taVNS vs current-discontinued sham | POD, cognition, biomarkers, safety | Protocol only | Zhou et al. protocol [46] | No | No efficacy results in protocol |
| Hao et al. vulnerable-brain-function protocol | ITMCTR2025002263 | Older adults undergoing abdominal surgery | Bilateral taVNS vs non-vagal active control | POD, cognition, biomarkers and function | Protocol only | Hao et al. protocol [47] | No | No efficacy results in protocol |

After de-duplication, scope assessment, and study-level linkage to completed efficacy reports, four unique protocol or registry-linked studies without published efficacy results were retained as directly relevant to perioperative taVNS and cognitive or delirium outcomes. Duplicate registry entries, records outside the prespecified perioperative cognitive-outcome scope, and records linked to completed efficacy publications were not included in this protocol summary.

# Supplementary Table S3. PRISMA 2020 reporting checklist and locations

The checklist is mapped to the systematic-review and exploratory meta-analysis component embedded within the broader narrative review.

| **Section / topic** | **Item** | **PRISMA 2020 checklist item** | **Location in the review** |
| --- | --- | --- | --- |
| TITLE | 1 | Identify the report as a systematic review. | Title |
| ABSTRACT | 2 | See the PRISMA 2020 for Abstracts checklist. | Single-paragraph abstract integrating background, methods, results, and conclusions. |
| INTRODUCTION | 3 | Describe the rationale for the review in the context of existing knowledge. | Section 1 |
| INTRODUCTION | 4 | Provide an explicit statement of the objective(s) or question(s) the review addresses. | Section 1.4 |
| METHODS | 5 | Specify inclusion and exclusion criteria and how studies were grouped for syntheses. | Sections 2.3 and 2.6 |
| METHODS | 6 | Specify all databases, registers, websites, organizations, reference lists, and other sources searched, and the date each was last searched. | Section 2.2 and Supplementary Methods S1, including registry cross-checking. |
| METHODS | 7 | Present the full search strategies for all databases, registers, and websites, including filters and limits. | Supplementary Methods S1.1–S1.4 and the registry cross-checking description after S1.4. |
| METHODS | 8 | Specify methods used to decide whether a study met the inclusion criteria, including reviewer independence and dispute resolution. | Section 2.4, Study selection. |
| METHODS | 9 | Specify methods used to collect data, including reviewer independence, author contact, and any automation tools. | Section 2.5. During revision, the corresponding authors of two included studies with key missing methodological or numerical information were contacted by email. No response had been received by the revision cutoff date. No automation tool was used as a reviewer. |
| METHODS | 10a | List and define all outcomes sought and whether all compatible results were collected. | Sections 2.3, 2.5, and 2.6. |
| METHODS | 10b | List and define all other variables sought and assumptions made about missing or unclear information. | Section 2.5: study design, population, stimulation parameters, comparator, follow-up, biomarkers, safety, and registration. |
| METHODS | 11 | Specify methods used to assess risk of bias, including tools, assessors, and any automation. | Section 2.7; Supplementary Table S4 presents outcome-specific RoB 2 signaling-question responses, supporting source information, domain judgments, and rationale. |
| METHODS | 12 | Specify the effect measures used for each outcome. | Section 2.6: risk ratios with 95% CIs for binary outcomes; mean differences for continuous outcomes when reported. |
| METHODS | 13a | Describe processes used to decide which studies were eligible for each synthesis. | Sections 2.6 and 2.8. |
| METHODS | 13b | Describe methods required to prepare data for presentation or synthesis. | Sections 2.5, 2.6, and 2.8; outcome families were separated and compatible event counts were standardized. |
| METHODS | 13c | Describe methods used to tabulate or visually display results. | Tables 1–4 and Figures 1–3. |
| METHODS | 13d | Describe methods used to synthesize results and justify model choice. | Section 2.8: common-effect inverse-variance synthesis, Paule–Mandel random-effects sensitivity analysis, and Hartung–Knapp interval sensitivity analysis. |
| METHODS | 13e | Describe methods used to explore possible causes of heterogeneity. | Sections 2.8 and 3.3–3.4; clinical and outcome-definition heterogeneity was examined descriptively because only two trials were pooled. |
| METHODS | 13f | Describe sensitivity analyses conducted to assess robustness. | Sections 2.8 and 3.4: Paule–Mandel random-effects and Hartung–Knapp interval sensitivity analyses. |
| METHODS | 14 | Describe methods used to assess risk of bias due to missing results. | Section 2.7: funnel plots and statistical tests were not performed because fewer than 10 studies were available. |
| METHODS | 15 | Describe methods used to assess certainty in the body of evidence. | Section 2.7; Supplementary Tables S5a–S5b. |
| RESULTS | 16a | Describe results of the search and selection process, ideally with a flow diagram. | Section 3.1 and Figure 1. |
| RESULTS | 16b | Cite studies that might appear eligible but were excluded and explain why. | No report was excluded after full-report assessment; title/abstract-stage adjudications and exclusion reasons are documented in the screening log. |
| RESULTS | 17 | Cite each included study and present its characteristics. | Sections 3.2–3.3 and Tables 1–2 |
| RESULTS | 18 | Present risk-of-bias assessments for each included study. | Section 3.7, Figure 3, and Supplementary Table S4. |
| RESULTS | 19 | For all outcomes, present summary statistics and effect estimates for each study. | Sections 3.4-3.6; Table 1; Figure 2. |
| RESULTS | 20a | For each synthesis, summarize contributing studies and risk of bias. | Sections 3.4–3.8; Table 1; Figure 3; Supplementary Table S4. |
| RESULTS | 20b | Present results of all statistical syntheses, including precision and heterogeneity. | Section 3.4 and Figure 2 |
| RESULTS | 20c | Present results of investigations of possible causes of heterogeneity. | Sections 3.3, 3.4, and 5.3. |
| RESULTS | 20d | Present results of sensitivity analyses. | Section 3.4: Paule–Mandel and Hartung–Knapp sensitivity results. |
| RESULTS | 21 | Present assessments of risk of bias due to missing results. | Formal assessment was not performed because fewer than 10 studies were available; see Sections 2.7 and 5.7. |
| RESULTS | 22 | Present certainty assessments for each outcome. | Section 3.8, Table 3, and Supplementary Tables S5a–S5b. |
| DISCUSSION | 23a | Provide a general interpretation of results in the context of other evidence. | Sections 5.1–5.4 |
| DISCUSSION | 23b | Discuss limitations of the evidence included in the review. | Sections 5.3–5.7 |
| DISCUSSION | 23c | Discuss limitations of the review processes used. | Section 5.7 |
| DISCUSSION | 23d | Discuss implications for practice, policy, and future research. | Sections 5.6 and 6, and Conclusions. |
| OTHER | 24a | Provide registration information, including register name and registration number, or state that the review was not registered. | Section 2.1 and the external registration record. |
| OTHER | 24b | Indicate where the review protocol can be accessed, or state that a protocol was not prepared. | Registration record and prespecified review methods. |
| OTHER | 24c | Describe and explain amendments to information provided at registration or in the protocol. | Sections 2.1 and 2.8. The PROSPERO registration prespecified separate analyses of dNCR and investigator-defined historical POCD. After study selection, a post hoc exploratory cross-construct synthesis of one dNCR trial and one traditional POCD trial was added to assess concordance in treatment effects across related early cognitive outcomes. No changes were made to eligibility criteria, search methods, or study selection. |
| OTHER | 25 | Describe sources of financial or non-financial support and the role of funders. | Funding |
| OTHER | 26 | Declare competing interests. | Conflict of Interest |
| OTHER | 27 | Report availability of data, code, forms, and other materials. | Data Availability and Supplementary Materials, including Supplementary Data S6 and Supplementary Code S7 |

# Supplementary Table S4. Detailed Cochrane RoB 2 judgments and supporting rationale

RoB 2 assessments are outcome-specific and use the standard signaling questions. Responses are Y (yes), PY (probably yes), PN (probably no), N (no), NA (not applicable), and NI (no information). NA indicates that a conditional question is not applicable under the official branching structure; NI means that available information is insufficient to answer an applicable question. Conditional questions not triggered by preceding responses are marked NA or omitted. Response options are question-specific; for question 3.2, the official options are NA/Y/PY/PN/N and do not include NI. Overall judgments follow RoB 2 decision rules; unavailable information is not treated as a negative response.

| **Study** | **Outcome** | **RoB 2 domain** | **Signaling question** | **Response (Y/PY/PN/N/NA/NI)** | **Exact supporting trial information** | **Source location** | **Domain judgment** | **Judgment rationale** |
| --- | --- | --- | --- | --- | --- | --- | --- | --- |
| Zhou et al., 2022 | dNCR at postoperative week 1 | Bias arising from the randomization process (1) | 1.1 Was the allocation sequence random? | Y | The article states that participants were allocated according to a random-number table. | Article p. 2422, Methods, Randomization and blinding. |  |  |
| Zhou et al., 2022 | dNCR at postoperative week 1 | Bias arising from the randomization process (1) | 1.2 Was the allocation sequence concealed until participants were enrolled and assigned to interventions? | NI | No method for concealing the upcoming assignment is described. | Article p. 2422, Methods, Randomization and blinding. |  |  |
| Zhou et al., 2022 | dNCR at postoperative week 1 | Bias arising from the randomization process (1) | 1.3 Did baseline differences between intervention groups suggest a problem with the randomization process? | PN | The report states demographic and intraoperative characteristics did not differ significantly; baseline characteristics are tabulated. | Article p. 2424, Results, Table 1; Supplementary Table S1. | Some concerns | The sequence was generated with a random-number table and baseline groups were balanced, but allocation concealment was not described. |
| Zhou et al., 2022 | dNCR at postoperative week 1 | Bias due to deviations from intended interventions (2) | 2.1 Were participants aware of their assigned intervention during the trial? | PN | The report states that patients and investigators were blinded and that opaque earmuffs covered the electrodes. | Article p. 2423, Methods, Randomization and blinding. |  |  |
| Zhou et al., 2022 | dNCR at postoperative week 1 | Bias due to deviations from intended interventions (2) | 2.2 Were carers and people delivering the interventions aware of participants' assigned intervention during the trial? | Y | A nurse placed the cymba-conchae active electrode or the earlobe/tail-of-helix sham clips; the placement identifies the assignment to the operator. | Article pp. 2422–2423, Methods, Randomization and blinding; Fig. 1. |  |  |
| Zhou et al., 2022 | dNCR at postoperative week 1 | Bias due to deviations from intended interventions (2) | 2.3 If Y/PY/NI to 2.1 or 2.2: Were there deviations from the intended intervention that arose because of the trial context? | NI | Three participants did not receive the assigned intervention (two early terminations; one combined anesthesia), but the reasons for early termination and whether the departures arose from trial context are not described. | Article p. 2424, Results, participant flow/Fig. 2. |  |  |
| Zhou et al., 2022 | dNCR at postoperative week 1 | Bias due to deviations from intended interventions (2) | 2.4 If Y/PY to 2.3: Were these deviations likely to have affected the outcome? | NA | The preceding response to 2.3 was NI; question 2.4 is triggered only by Y/PY. | Official conditional branch; Article p. 2424, Results, participant flow. |  |  |
| Zhou et al., 2022 | dNCR at postoperative week 1 | Bias due to deviations from intended interventions (2) | 2.5 If Y/PY/NI to 2.4: Were these deviations from intended intervention balanced between groups? | NA | Question 2.4 was not triggered; this question is conditional on Y/PY to 2.4. | Official conditional branch. |  |  |
| Zhou et al., 2022 | dNCR at postoperative week 1 | Bias due to deviations from intended interventions (2) | 2.6 Was an appropriate analysis used to estimate the effect of assignment to intervention? | Y | The report labels the analysis intention-to-treat and analyzes participants in their randomized groups; participants without week-1 testing are handled under missing outcome data. | Article p. 2424, Results and Statistical analysis; Fig. 2. |  |  |
| Zhou et al., 2022 | dNCR at postoperative week 1 | Bias due to deviations from intended interventions (2) | 2.7 If N/PN/NI to 2.6: Was there potential for a substantial impact (on the result) of the failure to analyse participants in the group to which they were randomized? | NA | Question 2.6 was answered Y, so the conditional question about impact of an inappropriate analysis was not triggered. | Official conditional branch. | Some concerns | Patients/investigators were described as blinded, but the nurse placing visibly different electrodes was likely aware; three assigned-treatment departures are reported and whether any arose from trial context is not stated. The assignment-group analysis was reported as ITT. |
| Zhou et al., 2022 | dNCR at postoperative week 1 | Bias due to missing outcome data (3) | 3.1 Were data for this outcome available for all, or nearly all, participants randomized? | Y | 119 of 124 randomized participants completed neuropsychological testing at one week; 60 taVNS and 59 sham participants contributed, with 22 observed dNCR events. | Article pp. 2424–2425, Results, Fig. 2 and dNCR outcome analysis. |  |  |
| Zhou et al., 2022 | dNCR at postoperative week 1 | Bias due to missing outcome data (3) | 3.2 If N/PN/NI to 3.1: Is there evidence that the result was not biased by missing outcome data? | NA | Question 3.1 was Y; the conditional missing-data follow-up was not triggered. | Official conditional branch. |  |  |
| Zhou et al., 2022 | dNCR at postoperative week 1 | Bias due to missing outcome data (3) | 3.3 If N/PN to 3.2: Could missingness in the outcome depend on its true value? | NA | Question 3.1 was Y; question 3.3 was not triggered. | Official conditional branch. |  |  |
| Zhou et al., 2022 | dNCR at postoperative week 1 | Bias due to missing outcome data (3) | 3.4 If Y/PY/NI to 3.3: Is it likely that missingness in the outcome depended on its true value? | NA | Question 3.1 was Y; question 3.4 was not triggered. | Official conditional branch. | Low | Week-1 testing was available for 119/124 randomized participants; five missing outcomes are fewer than the 22 observed dNCR events. |
| Zhou et al., 2022 | dNCR at postoperative week 1 | Bias in measurement of the outcome (4) | 4.1 Was the method of measuring the outcome inappropriate? | N | The outcome used a neuropsychological battery (RAVLT, Trail Making, Digit Symbol Substitution, and Stroop) with a prespecified reliable-change approach against matched nonsurgical controls. | Article pp. 2423–2424, Methods, Outcome analysis; Results. |  |  |
| Zhou et al., 2022 | dNCR at postoperative week 1 | Bias in measurement of the outcome (4) | 4.2 Could measurement or ascertainment of the outcome have differed between intervention groups? | N | The same battery, baseline testing, and one-week follow-up were used in both randomized groups. | Article pp. 2423–2424, Methods, Outcome analysis; Results. |  |  |
| Zhou et al., 2022 | dNCR at postoperative week 1 | Bias in measurement of the outcome (4) | 4.3 If N/PN/NI to 4.1 and 4.2: Were outcome assessors aware of the intervention received by study participants? | PN | Patients and investigators were stated to be blinded; the article does not separately identify all outcome assessors. | Article p. 2423, Methods, Randomization and blinding. |  |  |
| Zhou et al., 2022 | dNCR at postoperative week 1 | Bias in measurement of the outcome (4) | 4.4 If Y/PY/NI to 4.3: Could assessment of the outcome have been influenced by knowledge of the intervention received? | NA | Question 4.3 was PN; influence of assessor awareness was not applicable. | Official conditional branch. |  |  |
| Zhou et al., 2022 | dNCR at postoperative week 1 | Bias in measurement of the outcome (4) | 4.5 If Y/PY/NI to 4.4: Is it likely that assessment of the outcome was influenced by knowledge of the intervention received? | NA | Question 4.4 was not triggered. | Official conditional branch. | Low | A neuropsychological battery appropriate to the reported dNCR endpoint was administered at the same time points, and the report states patients/investigators were blinded. |
| Zhou et al., 2022 | dNCR at postoperative week 1 | Bias in selection of the reported result (5) | 5.1 Were the data that produced this result analysed in accordance with a pre-specified analysis plan that was finalized before unblinded outcome data were available for analysis? | NI | ChiCTR2100044905 is reported, but a sufficiently detailed dated statistical analysis plan was not available in the supplied primary-source set. | Article p. 2422, Methods; trial registration record not supplied. |  |  |
| Zhou et al., 2022 | dNCR at postoperative week 1 | Bias in selection of the reported result (5) | 5.2 Is the numerical result being assessed likely to have been selected, on the basis of the results, from multiple eligible outcome measurements (e.g. scales, definitions, time points) within the outcome domain? | PN | The dNCR definition and one-week assessment are explicitly described; no source evidence indicates selection among multiple eligible definitions based on results. | Article pp. 2423–2424, Methods, Outcome analysis; Results. |  |  |
| Zhou et al., 2022 | dNCR at postoperative week 1 | Bias in selection of the reported result (5) | 5.3 Is the numerical result being assessed likely to have been selected, on the basis of the results, from multiple eligible analyses of the data? | NI | The paper reports an analysis section but provides no dated plan for comparison of all eligible analyses of the neuropsychological battery/composite outcome. | Article pp. 2423–2424, Methods, Statistical analysis and Outcome analysis. | Some concerns | The trial registration is reported, but a dated statistical analysis plan could not be compared with the reported result; the study defines a specific week-1 endpoint, with no evidence of result-driven selection, while analytic multiplicity cannot be excluded. |
| Zhou et al., 2022 | dNCR at postoperative week 1 | Overall judgment | Overall RoB 2 judgment for the specified outcome |  | Outcome-specific ratings follow the official domain decision algorithms; no manual override was applied. | All domain locations cited above. | Some concerns | Overall Some concerns because at least one domain was Some concerns (Bias arising from the randomization process: Some concerns; Bias due to deviations from intended interventions: Some concerns; Bias due to missing outcome data: Low; Bias in measurement of the outcome: Low; Bias in selection of the reported result: Some concerns); no domain was rated High. |
| Shi et al., 2026 | POD on postoperative days 1–3 | Bias arising from the randomization process (1) | 1.1 Was the allocation sequence random? | Y | Participants were centrally randomized 1:1 using a computer-generated random-number table. | Article PDF p. 3 (article p. 2), Methods, Randomization and Blinding. |  |  |
| Shi et al., 2026 | POD on postoperative days 1–3 | Bias arising from the randomization process (1) | 1.2 Was the allocation sequence concealed until participants were enrolled and assigned to interventions? | PY | Allocation was centrally generated and concealed in opaque envelopes until arrival in the operating room; sequence numbering/sealing details are not fully stated. | Article PDF p. 3 (article p. 2), Methods, Randomization and Blinding. |  |  |
| Shi et al., 2026 | POD on postoperative days 1–3 | Bias arising from the randomization process (1) | 1.3 Did baseline differences between intervention groups suggest a problem with the randomization process? | PN | Only ASA classification differed at baseline (P = 0.022) among numerous baseline characteristics; a small number of nominal P < 0.05 differences is compatible with chance under RoB 2 guidance. | Article PDF pp. 5–7 (article pp. 4–6), Table 1. | Low | Computer-generated random numbers and central allocation with opaque envelopes were reported; the isolated ASA imbalance is compatible with chance under RoB 2 guidance. |
| Shi et al., 2026 | POD on postoperative days 1–3 | Bias due to deviations from intended interventions (2) | 2.1 Were participants aware of their assigned intervention during the trial? | PY | The study is described as double blind; participant-specific masking and a blinding-success test are not separately reported. | Article PDF pp. 3–4, Methods, Randomization and Blinding. |  |  |
| Shi et al., 2026 | POD on postoperative days 1–3 | Bias due to deviations from intended interventions (2) | 2.2 Were carers and people delivering the interventions aware of participants' assigned intervention during the trial? | Y | The investigator who revealed the envelope in the operating room administered stimulation and therefore knew the assigned site; assessors, data processors, surgeons, anesthesiologists, and nurses were masked. | Article PDF pp. 3–4, Methods, Randomization and Blinding. |  |  |
| Shi et al., 2026 | POD on postoperative days 1–3 | Bias due to deviations from intended interventions (2) | 2.3 If Y/PY/NI to 2.1 or 2.2: Were there deviations from the intended intervention that arose because of the trial context? | PN | All 150 participants completed the trial; no departures from the assigned single-session protocol or trial-context co-interventions are reported. | Article PDF pp. 5–6, Results, participant flow; Methods, Intervention. |  |  |
| Shi et al., 2026 | POD on postoperative days 1–3 | Bias due to deviations from intended interventions (2) | 2.4 If Y/PY to 2.3: Were these deviations likely to have affected the outcome? | NA | Question 2.3 was PN; question 2.4 is conditional on Y/PY. | Official conditional branch. |  |  |
| Shi et al., 2026 | POD on postoperative days 1–3 | Bias due to deviations from intended interventions (2) | 2.5 If Y/PY/NI to 2.4: Were these deviations from intended intervention balanced between groups? | NA | Question 2.4 was not triggered. | Official conditional branch. |  |  |
| Shi et al., 2026 | POD on postoperative days 1–3 | Bias due to deviations from intended interventions (2) | 2.6 Was an appropriate analysis used to estimate the effect of assignment to intervention? | Y | All 150 randomized participants were analyzed by assigned group using the intention-to-treat principle. | Article PDF p. 5, Methods, Statistical Analysis; Results, participant flow. |  |  |
| Shi et al., 2026 | POD on postoperative days 1–3 | Bias due to deviations from intended interventions (2) | 2.7 If N/PN/NI to 2.6: Was there potential for a substantial impact (on the result) of the failure to analyse participants in the group to which they were randomized? | NA | Question 2.6 was Y; the conditional question about impact of inappropriate analysis was not triggered. | Official conditional branch. | Low | The trial was described as double blind; intervention administrators opened allocation and delivered site-specific stimulation, but all participants completed the trial and no trial-context deviations were reported. An appropriate ITT analysis was used. |
| Shi et al., 2026 | POD on postoperative days 1–3 | Bias due to missing outcome data (3) | 3.1 Were data for this outcome available for all, or nearly all, participants randomized? | Y | The Results state that all 150 randomized participants completed the trial; no missing primary POD outcomes were reported. | Article PDF p. 5 (article p. 4), Results, participant flow; Methods, Statistical Analysis. |  |  |
| Shi et al., 2026 | POD on postoperative days 1–3 | Bias due to missing outcome data (3) | 3.2 If N/PN/NI to 3.1: Is there evidence that the result was not biased by missing outcome data? | NA | Question 3.1 was Y. | Official conditional branch. |  |  |
| Shi et al., 2026 | POD on postoperative days 1–3 | Bias due to missing outcome data (3) | 3.3 If N/PN to 3.2: Could missingness in the outcome depend on its true value? | NA | Question 3.1 was Y. | Official conditional branch. |  |  |
| Shi et al., 2026 | POD on postoperative days 1–3 | Bias due to missing outcome data (3) | 3.4 If Y/PY/NI to 3.3: Is it likely that missingness in the outcome depended on its true value? | NA | Question 3.1 was Y. | Official conditional branch. | Low | Primary-outcome data were reported for all 150 randomized participants; no primary-outcome imputation was required. |
| Shi et al., 2026 | POD on postoperative days 1–3 | Bias in measurement of the outcome (4) | 4.1 Was the method of measuring the outcome inappropriate? | N | CAM/CAM-ICU are appropriate instruments for delirium assessment. | Article PDF pp. 4–5, Methods, Outcome measures and data collection. |  |  |
| Shi et al., 2026 | POD on postoperative days 1–3 | Bias in measurement of the outcome (4) | 4.2 Could measurement or ascertainment of the outcome have differed between intervention groups? | N | Both groups had the same assessment schedule (twice daily for three postoperative days) and delirium criteria. | Article PDF pp. 4–5, Methods, Outcome measures and data collection. |  |  |
| Shi et al., 2026 | POD on postoperative days 1–3 | Bias in measurement of the outcome (4) | 4.3 If N/PN/NI to 4.1 and 4.2: Were outcome assessors aware of the intervention received by study participants? | PN | Outcome assessors and researchers collecting/processing outcome data were reported masked to allocation. | Article PDF pp. 3–4, Methods, Randomization and Blinding. |  |  |
| Shi et al., 2026 | POD on postoperative days 1–3 | Bias in measurement of the outcome (4) | 4.4 If Y/PY/NI to 4.3: Could assessment of the outcome have been influenced by knowledge of the intervention received? | NA | Question 4.3 was PN. | Official conditional branch. |  |  |
| Shi et al., 2026 | POD on postoperative days 1–3 | Bias in measurement of the outcome (4) | 4.5 If Y/PY/NI to 4.4: Is it likely that assessment of the outcome was influenced by knowledge of the intervention received? | NA | Question 4.4 was not triggered. | Official conditional branch. | Low | POD was assessed with validated CAM/CAM-ICU instruments at repeated scheduled assessments by trained masked assessors. |
| Shi et al., 2026 | POD on postoperative days 1–3 | Bias in selection of the reported result (5) | 5.1 Were the data that produced this result analysed in accordance with a pre-specified analysis plan that was finalized before unblinded outcome data were available for analysis? | NI | ChiCTR2400090660 is reported, but no dated statistical analysis plan was available in the supplied primary-source set. | Article PDF p. 3, Methods; registry history/SAP not supplied. |  |  |
| Shi et al., 2026 | POD on postoperative days 1–3 | Bias in selection of the reported result (5) | 5.2 Is the numerical result being assessed likely to have been selected, on the basis of the results, from multiple eligible outcome measurements (e.g. scales, definitions, time points) within the outcome domain? | PN | POD incidence within three days is identified as the primary endpoint and cumulative and day-specific results are reported; there is no evidence of result-driven selection among measurements. | Article PDF pp. 5–8, Methods and Results, Tables 1–2/Figures 2–3. |  |  |
| Shi et al., 2026 | POD on postoperative days 1–3 | Bias in selection of the reported result (5) | 5.3 Is the numerical result being assessed likely to have been selected, on the basis of the results, from multiple eligible analyses of the data? | NI | Subgroup and mediation analyses were also conducted, but the analysis plan needed to compare all eligible analyses with the reported effect was unavailable. | Article PDF pp. 5–6, Methods, Statistical Analysis; Results, subgroup and mediation analyses. | Some concerns | The primary POD outcome and repeated assessment window are reported, but a dated, sufficiently detailed analysis plan was not available for comparison with subgroup and mediation analyses. |
| Shi et al., 2026 | POD on postoperative days 1–3 | Overall judgment | Overall RoB 2 judgment for the specified outcome |  | Outcome-specific ratings follow the official domain decision algorithms; no manual override was applied. | All domain locations cited above. | Some concerns | Overall Some concerns because at least one domain was Some concerns (Bias arising from the randomization process: Low; Bias due to deviations from intended interventions: Low; Bias due to missing outcome data: Low; Bias in measurement of the outcome: Low; Bias in selection of the reported result: Some concerns); no domain was rated High. |
| Li et al., 2026 | Investigator-defined early POCD (MoCA decrease ≥2 points from baseline to POD7) | Bias arising from the randomization process (1) | 1.1 Was the allocation sequence random? | Y | Random numbers were generated in SPSS 25.0 at a 1:1 ratio. | Article p. 661, Methods, Randomization and Blinding. |  |  |
| Li et al., 2026 | Investigator-defined early POCD (MoCA decrease ≥2 points from baseline to POD7) | Bias arising from the randomization process (1) | 1.2 Was the allocation sequence concealed until participants were enrolled and assigned to interventions? | NI | No concealment method for the generated sequence is described. | Article p. 661, Methods, Randomization and Blinding. |  |  |
| Li et al., 2026 | Investigator-defined early POCD (MoCA decrease ≥2 points from baseline to POD7) | Bias arising from the randomization process (1) | 1.3 Did baseline differences between intervention groups suggest a problem with the randomization process? | PN | The analyzed groups had no statistically significant baseline differences in reported characteristics. | Article pp. 662–663, Results, Table 1. | Some concerns | Random-number generation was reported and no important baseline imbalance was identified, but allocation concealment was not described. |
| Li et al., 2026 | Investigator-defined early POCD (MoCA decrease ≥2 points from baseline to POD7) | Bias due to deviations from intended interventions (2) | 2.1 Were participants aware of their assigned intervention during the trial? | NI | The paper does not state whether participants were masked; the comparator used 0 mA. | Article p. 661, Methods, Randomization and Blinding; Study Interventions. |  |  |
| Li et al., 2026 | Investigator-defined early POCD (MoCA decrease ≥2 points from baseline to POD7) | Bias due to deviations from intended interventions (2) | 2.2 Were carers and people delivering the interventions aware of participants' assigned intervention during the trial? | NI | The report identifies an intervention operator but does not state whether the person delivering stimulation knew assignment. | Article p. 661, Methods, Study Interventions. |  |  |
| Li et al., 2026 | Investigator-defined early POCD (MoCA decrease ≥2 points from baseline to POD7) | Bias due to deviations from intended interventions (2) | 2.3 If Y/PY/NI to 2.1 or 2.2: Were there deviations from the intended intervention that arose because of the trial context? | NI | Two active-arm participants were withdrawn from the analysis, including one who refused postoperative stimulation; the report does not establish whether the refusal/exclusion arose because of trial context. | Article p. 662, Results, Patient Characteristics/Fig. 1. |  |  |
| Li et al., 2026 | Investigator-defined early POCD (MoCA decrease ≥2 points from baseline to POD7) | Bias due to deviations from intended interventions (2) | 2.4 If Y/PY to 2.3: Were these deviations likely to have affected the outcome? | NA | Question 2.3 was NI; question 2.4 is conditional on Y/PY. | Official conditional branch. |  |  |
| Li et al., 2026 | Investigator-defined early POCD (MoCA decrease ≥2 points from baseline to POD7) | Bias due to deviations from intended interventions (2) | 2.5 If Y/PY/NI to 2.4: Were these deviations from intended intervention balanced between groups? | NA | Question 2.4 was not triggered. | Official conditional branch. |  |  |
| Li et al., 2026 | Investigator-defined early POCD (MoCA decrease ≥2 points from baseline to POD7) | Bias due to deviations from intended interventions (2) | 2.6 Was an appropriate analysis used to estimate the effect of assignment to intervention? | PN | The primary analysis excluded two randomized active-arm participants (one procedure cancellation and one refusal of postoperative stimulation); this is not a full ITT analysis retaining all randomized participants in assigned groups. | Article p. 662, Results, Patient Characteristics/Fig. 1. |  |  |
| Li et al., 2026 | Investigator-defined early POCD (MoCA decrease ≥2 points from baseline to POD7) | Bias due to deviations from intended interventions (2) | 2.7 If N/PN/NI to 2.6: Was there potential for a substantial impact (on the result) of the failure to analyse participants in the group to which they were randomized? | PN | Only 2/103 participants were excluded and 22 POCD events were observed; the report does not suggest that the small exclusions could have a substantial impact on the result. | Article p. 662, Results, Patient Characteristics and POCD outcome. | Some concerns | Participant and intervention-provider masking were not described, and one active-arm participant refused postoperative stimulation. Two randomized active-arm participants were excluded from the primary analysis; this is not a full assignment-group analysis, although the number excluded was small. |
| Li et al., 2026 | Investigator-defined early POCD (MoCA decrease ≥2 points from baseline to POD7) | Bias due to missing outcome data (3) | 3.1 Were data for this outcome available for all, or nearly all, participants randomized? | Y | Primary analysis included 101/103 randomized participants (49/51 active and 52/52 sham); 2 missing outcomes versus 22 observed POCD events. | Article p. 662, Results, Patient Characteristics/Fig. 1 and POCD analysis. |  |  |
| Li et al., 2026 | Investigator-defined early POCD (MoCA decrease ≥2 points from baseline to POD7) | Bias due to missing outcome data (3) | 3.2 If N/PN/NI to 3.1: Is there evidence that the result was not biased by missing outcome data? | NA | Question 3.1 was Y. | Official conditional branch. |  |  |
| Li et al., 2026 | Investigator-defined early POCD (MoCA decrease ≥2 points from baseline to POD7) | Bias due to missing outcome data (3) | 3.3 If N/PN to 3.2: Could missingness in the outcome depend on its true value? | NA | Question 3.1 was Y. | Official conditional branch. |  |  |
| Li et al., 2026 | Investigator-defined early POCD (MoCA decrease ≥2 points from baseline to POD7) | Bias due to missing outcome data (3) | 3.4 If Y/PY/NI to 3.3: Is it likely that missingness in the outcome depended on its true value? | NA | Question 3.1 was Y. | Official conditional branch. | Low | Primary cognitive data were available for 101/103 randomized participants, and the two missing records were fewer than the 22 observed POCD events. |
| Li et al., 2026 | Investigator-defined early POCD (MoCA decrease ≥2 points from baseline to POD7) | Bias in measurement of the outcome (4) | 4.1 Was the method of measuring the outcome inappropriate? | N | MoCA is an established cognitive screening measure appropriate for the reported investigator-defined score-based outcome; it is not interpreted as a consensus PND diagnostic instrument. | Article pp. 661–662, Methods, Outcome Measures; Results. |  |  |
| Li et al., 2026 | Investigator-defined early POCD (MoCA decrease ≥2 points from baseline to POD7) | Bias in measurement of the outcome (4) | 4.2 Could measurement or ascertainment of the outcome have differed between intervention groups? | N | MoCA testing was scheduled at baseline and POD1, POD3, and POD7 in both groups. | Article pp. 661–662, Methods, Outcome Measures/Data Collection. |  |  |
| Li et al., 2026 | Investigator-defined early POCD (MoCA decrease ≥2 points from baseline to POD7) | Bias in measurement of the outcome (4) | 4.3 If N/PN/NI to 4.1 and 4.2: Were outcome assessors aware of the intervention received by study participants? | PN | Investigators responsible for pre/postoperative assessment and data collection were stated to be blinded. | Article p. 661, Methods, Randomization and Blinding. |  |  |
| Li et al., 2026 | Investigator-defined early POCD (MoCA decrease ≥2 points from baseline to POD7) | Bias in measurement of the outcome (4) | 4.4 If Y/PY/NI to 4.3: Could assessment of the outcome have been influenced by knowledge of the intervention received? | NA | Question 4.3 was PN. | Official conditional branch. |  |  |
| Li et al., 2026 | Investigator-defined early POCD (MoCA decrease ≥2 points from baseline to POD7) | Bias in measurement of the outcome (4) | 4.5 If Y/PY/NI to 4.4: Is it likely that assessment of the outcome was influenced by knowledge of the intervention received? | NA | Question 4.4 was not triggered. | Official conditional branch. | Low | MoCA was measured at the same baseline and postoperative time points by blinded assessment/data-collection investigators. The score-based investigator definition is not a consensus dNCR diagnosis, which is an interpretive limitation rather than evidence of differential measurement. |
| Li et al., 2026 | Investigator-defined early POCD (MoCA decrease ≥2 points from baseline to POD7) | Bias in selection of the reported result (5) | 5.1 Were the data that produced this result analysed in accordance with a pre-specified analysis plan that was finalized before unblinded outcome data were available for analysis? | NI | The article calls ChiCTR2500115495 retrospectively registered but also states recruitment began after trial registration; no dated SAP/protocol is available to resolve the inconsistency. | Article p. 661, Methods, study registration and recruitment; registry record not supplied. |  |  |
| Li et al., 2026 | Investigator-defined early POCD (MoCA decrease ≥2 points from baseline to POD7) | Bias in selection of the reported result (5) | 5.2 Is the numerical result being assessed likely to have been selected, on the basis of the results, from multiple eligible outcome measurements (e.g. scales, definitions, time points) within the outcome domain? | NI | MoCA was assessed at multiple time points and the binary POCD cutoff is specified in the paper, but no pre-result plan permits assessment of selection among all eligible outcome measurements. | Article pp. 661–662, Methods, Outcome Measures; Results. |  |  |
| Li et al., 2026 | Investigator-defined early POCD (MoCA decrease ≥2 points from baseline to POD7) | Bias in selection of the reported result (5) | 5.3 Is the numerical result being assessed likely to have been selected, on the basis of the results, from multiple eligible analyses of the data? | NI | The report includes binary POCD, serial MoCA, and other analyses; a dated SAP is unavailable, but there is no direct evidence that the result was selected because of its observed value. | Article pp. 661–664, Methods/Results; registry timing discrepancy noted above. | Some concerns | The study reports retrospective registration and no dated prespecified analysis plan was available; the article also contains inconsistent statements about whether recruitment began before or after registration. Multiple time points and analyses are reported, but result-driven selection is not established. |
| Li et al., 2026 | Investigator-defined early POCD (MoCA decrease ≥2 points from baseline to POD7) | Overall judgment | Overall RoB 2 judgment for the specified outcome |  | Outcome-specific ratings follow the official domain decision algorithms; no manual override was applied. | All domain locations cited above. | Some concerns | Overall Some concerns because at least one domain was Some concerns (Bias arising from the randomization process: Some concerns; Bias due to deviations from intended interventions: Some concerns; Bias due to missing outcome data: Low; Bias in measurement of the outcome: Low; Bias in selection of the reported result: Some concerns); no domain was rated High. |
| Ma et al., 2026 | Longitudinal MoCA trajectory through postoperative month 3 | Bias arising from the randomization process (1) | 1.1 Was the allocation sequence random? | NI | The article says participants were randomly assigned but does not describe how the allocation sequence was generated. | PDF p. 2, Section 2.3, Randomization, blinding, and intervention. |  |  |
| Ma et al., 2026 | Longitudinal MoCA trajectory through postoperative month 3 | Bias arising from the randomization process (1) | 1.2 Was the allocation sequence concealed until participants were enrolled and assigned to interventions? | Y | Sequentially numbered, sealed opaque envelopes were prepared by an independent coordinator not involved in recruitment, intervention delivery, or outcome assessment; the coordinator prepared the active/sham device. | PDF p. 2, Section 2.3, Randomization, blinding, and intervention. |  |  |
| Ma et al., 2026 | Longitudinal MoCA trajectory through postoperative month 3 | Bias arising from the randomization process (1) | 1.3 Did baseline differences between intervention groups suggest a problem with the randomization process? | PN | Age differed at P = 0.04, while other baseline characteristics were reported comparable; an isolated nominal baseline difference among multiple characteristics is compatible with chance under RoB 2 guidance. | PDF pp. 3–4, Results, Table 1. | Some concerns | Sequentially numbered sealed opaque envelopes prepared by an independent coordinator support concealment, and the isolated baseline age difference is compatible with chance; the random-sequence generation method itself is not reported. |
| Ma et al., 2026 | Longitudinal MoCA trajectory through postoperative month 3 | Bias due to deviations from intended interventions (2) | 2.1 Were participants aware of their assigned intervention during the trial? | PY | The article describes a double-blind trial and the sham device had the same appearance/interface, but participant-specific masking and a formal blinding-success assessment are not reported. | PDF pp. 2–3, Sections 2.1 and 2.3; Figure 2. |  |  |
| Ma et al., 2026 | Longitudinal MoCA trajectory through postoperative month 3 | Bias due to deviations from intended interventions (2) | 2.2 Were carers and people delivering the interventions aware of participants' assigned intervention during the trial? | NI | Outcome assessors were explicitly unaware; awareness of the personnel delivering stimulation was not stated. | PDF p. 2, Section 2.3, Randomization, blinding, and intervention. |  |  |
| Ma et al., 2026 | Longitudinal MoCA trajectory through postoperative month 3 | Bias due to deviations from intended interventions (2) | 2.3 If Y/PY/NI to 2.1 or 2.2: Were there deviations from the intended intervention that arose because of the trial context? | NI | Four active-arm participants withdrew consent after randomization and before intervention, but the report does not explain whether this arose because of the trial context. | PDF p. 3, Results, participant flow/Figure 1. |  |  |
| Ma et al., 2026 | Longitudinal MoCA trajectory through postoperative month 3 | Bias due to deviations from intended interventions (2) | 2.4 If Y/PY to 2.3: Were these deviations likely to have affected the outcome? | NA | Question 2.3 was NI; question 2.4 is conditional on Y/PY. | Official conditional branch. |  |  |
| Ma et al., 2026 | Longitudinal MoCA trajectory through postoperative month 3 | Bias due to deviations from intended interventions (2) | 2.5 If Y/PY/NI to 2.4: Were these deviations from intended intervention balanced between groups? | NA | Question 2.4 was not triggered. | Official conditional branch. |  |  |
| Ma et al., 2026 | Longitudinal MoCA trajectory through postoperative month 3 | Bias due to deviations from intended interventions (2) | 2.6 Was an appropriate analysis used to estimate the effect of assignment to intervention? | Y | The main repeated-measures analysis used the randomized groups among participants with observed data; an ITT multiple-imputation sensitivity analysis included all 103 and yielded consistent inferences. | PDF pp. 2–3, Sections 2.5 and Results; Supplementary Methods/Table S10 referenced in article. |  |  |
| Ma et al., 2026 | Longitudinal MoCA trajectory through postoperative month 3 | Bias due to deviations from intended interventions (2) | 2.7 If N/PN/NI to 2.6: Was there potential for a substantial impact (on the result) of the failure to analyse participants in the group to which they were randomized? | NA | Question 2.6 was Y; the conditional question on impact of an inappropriate analysis was not triggered. | Official conditional branch. | Some concerns | The trial is described as double blind and assessors were masked, but participant/provider-specific masking and trial-context deviations were not fully documented. A complete-case primary analysis was supplemented by an ITT multiple-imputation sensitivity analysis. |
| Ma et al., 2026 | Longitudinal MoCA trajectory through postoperative month 3 | Bias due to missing outcome data (3) | 3.1 Were data for this outcome available for all, or nearly all, participants randomized? | PY | The complete-case longitudinal outcome included 99/103 randomized participants (49/53 active, 50/50 control); the four active-arm withdrawals occurred before intervention. The article also reports an ITT multiple-imputation sensitivity analysis with n=103 and consistent inferences. | PDF pp. 3–4, Results, participant flow; Section 2.5 and Supplementary Methods/Table S10 referenced in article. |  |  |
| Ma et al., 2026 | Longitudinal MoCA trajectory through postoperative month 3 | Bias due to missing outcome data (3) | 3.2 If N/PN/NI to 3.1: Is there evidence that the result was not biased by missing outcome data? | NA | Question 3.1 was PY; the conditional missing-data follow-up was not triggered. | Official conditional branch. |  |  |
| Ma et al., 2026 | Longitudinal MoCA trajectory through postoperative month 3 | Bias due to missing outcome data (3) | 3.3 If N/PN to 3.2: Could missingness in the outcome depend on its true value? | NA | Question 3.1 was PY. | Official conditional branch. |  |  |
| Ma et al., 2026 | Longitudinal MoCA trajectory through postoperative month 3 | Bias due to missing outcome data (3) | 3.4 If Y/PY/NI to 3.3: Is it likely that missingness in the outcome depended on its true value? | NA | Question 3.1 was PY. | Official conditional branch. | Low | Complete-case MoCA data were available for 99/103 participants (96.1%); although all four missing participants were assigned to taVNS, the article reports a consistent ITT multiple-imputation sensitivity analysis including all randomized participants. |
| Ma et al., 2026 | Longitudinal MoCA trajectory through postoperative month 3 | Bias in measurement of the outcome (4) | 4.1 Was the method of measuring the outcome inappropriate? | N | MoCA is appropriate for measurement of the reported serial cognitive-score trajectory; the result is not treated as a formal PND diagnosis. | PDF pp. 2–4, Section 2.4, Outcome measures; Table 2. |  |  |
| Ma et al., 2026 | Longitudinal MoCA trajectory through postoperative month 3 | Bias in measurement of the outcome (4) | 4.2 Could measurement or ascertainment of the outcome have differed between intervention groups? | N | The same MoCA schedule was used at baseline, POD1, POD7, month 1, and month 3 in both groups. | PDF pp. 2–4, Sections 2.3–2.4; Table 2. |  |  |
| Ma et al., 2026 | Longitudinal MoCA trajectory through postoperative month 3 | Bias in measurement of the outcome (4) | 4.3 If N/PN/NI to 4.1 and 4.2: Were outcome assessors aware of the intervention received by study participants? | PN | The article states outcome assessors remained unaware of group allocation throughout. | PDF p. 2, Section 2.3, Randomization, blinding, and intervention. |  |  |
| Ma et al., 2026 | Longitudinal MoCA trajectory through postoperative month 3 | Bias in measurement of the outcome (4) | 4.4 If Y/PY/NI to 4.3: Could assessment of the outcome have been influenced by knowledge of the intervention received? | NA | Question 4.3 was PN. | Official conditional branch. |  |  |
| Ma et al., 2026 | Longitudinal MoCA trajectory through postoperative month 3 | Bias in measurement of the outcome (4) | 4.5 If Y/PY/NI to 4.4: Is it likely that assessment of the outcome was influenced by knowledge of the intervention received? | NA | Question 4.4 was not triggered. | Official conditional branch. | Low | Serial MoCA is appropriate for the reported cognitive-score trajectory; the same schedule was used in each group and outcome assessors were explicitly masked. |
| Ma et al., 2026 | Longitudinal MoCA trajectory through postoperative month 3 | Bias in selection of the reported result (5) | 5.1 Were the data that produced this result analysed in accordance with a pre-specified analysis plan that was finalized before unblinded outcome data were available for analysis? | NI | ChiCTR2400094874 is reported, but a dated SAP specifying the repeated-measures model, adjustment set, and time-point hierarchy was not available in the supplied primary-source set. | PDF pp. 1–2, trial registration and Section 2.3; registry history/SAP not supplied. |  |  |
| Ma et al., 2026 | Longitudinal MoCA trajectory through postoperative month 3 | Bias in selection of the reported result (5) | 5.2 Is the numerical result being assessed likely to have been selected, on the basis of the results, from multiple eligible outcome measurements (e.g. scales, definitions, time points) within the outcome domain? | PN | The paper reports MoCA at baseline and all four postoperative time points rather than reporting only a selected favorable time point. | PDF pp. 2–4, Sections 2.4/Results; Table 2. |  |  |
| Ma et al., 2026 | Longitudinal MoCA trajectory through postoperative month 3 | Bias in selection of the reported result (5) | 5.3 Is the numerical result being assessed likely to have been selected, on the basis of the results, from multiple eligible analyses of the data? | NI | Several repeated-measures, age-adjusted, and sensitivity analyses are reported, but the unavailable dated SAP prevents comparison of all eligible analyses; there is no direct evidence of result-driven selection. | PDF pp. 2–5, Sections 2.5 and 3.2; Tables 2 and Supplementary Tables S1–S2 referenced. | Some concerns | MoCA is identified as the primary cognitive outcome and all scheduled time points are reported, but a dated prespecified analysis plan is not available to evaluate selection among multiple repeated-measures and adjusted analyses. |
| Ma et al., 2026 | Longitudinal MoCA trajectory through postoperative month 3 | Overall judgment | Overall RoB 2 judgment for the specified outcome |  | Outcome-specific ratings follow the official domain decision algorithms; no manual override was applied. | All domain locations cited above. | Some concerns | Overall Some concerns because at least one domain was Some concerns (Bias arising from the randomization process: Some concerns; Bias due to deviations from intended interventions: Some concerns; Bias due to missing outcome data: Low; Bias in measurement of the outcome: Low; Bias in selection of the reported result: Some concerns); no domain was rated High. |

*Abbreviations: dNCR, delayed neurocognitive recovery; MoCA, Montreal Cognitive Assessment; POD, postoperative delirium; POCD, postoperative cognitive dysfunction.*

# Supplementary Table S5a. Full GRADE evidence profiles

Certainty starts at high for randomized trials and is downgraded for serious or very serious concerns across the five GRADE domains. No upgrading criteria were applied.

| **Outcome** | **Studies / participants** | **Risk of bias** | **Inconsistency** | **Indirectness** | **Imprecision** | **Publication bias** | **Overall certainty** |
| --- | --- | --- | --- | --- | --- | --- | --- |
| Post hoc exploratory cross-construct synthesis of dNCR and investigator-defined traditional POCD | 2 RCTs / 220 | Serious (-1)ᵃ | Not serious; assessment limited by only two studies | Serious (-1)ᵇ | Serious (-1)ᶜ | Not assessable | Very low |
| Postoperative delirium | 1 RCT / 150 | Serious (-1)ᵈ | Not assessable; no downgrade | Not serious | Serious (-1)ᵉ | Not assessable | Low |
| Longitudinal MoCA trajectory | 1 RCT / 99 | Serious (-1)ᶠ | Not assessable; no downgrade | Serious (-1)ᵍ | Serious (-1)ʰ | Not assessable | Very low |

## Supplementary Table S5b. Summary of findings

| **Outcome and time frame** | **Participants (studies)** | **Relative / continuous effect** | **Control risk or mean** | **Absolute effect with taVNS** | **Certainty** | **Interpretation** |
| --- | --- | --- | --- | --- | --- | --- |
| Post hoc exploratory cross-construct synthesis of dNCR and investigator-defined traditional POCD, within the first postoperative week | 220 (2 RCTs) | RR 0.34 (95% CI 0.18-0.64) | 297 per 1,000 (33/111) | 101 per 1,000 (54-190); 196 fewer per 1,000 (107-244 fewer) | Very low | The pooled estimate is highly uncertain and represents an exploratory cross-construct synthesis, not an effect estimate for one standardized endpoint. |
| Postoperative delirium during postoperative days 1-3 | 150 (1 RCT) | RR 0.47 (95% CI 0.23-0.98) | 253 per 1,000 (19/75) | 119 per 1,000 (58-248); 134 fewer per 1,000 (5-195 fewer) | Low | taVNS may reduce POD, but the result derives from a single trial with few events and a confidence interval close to no effect. |
| Longitudinal MoCA trajectory, postoperative month 1 and month 3 | 99 (1 RCT) | MD +2.06 points at month 1; MD +2.97 points at month 3 | Study-specific sham-group trajectory | More favorable adjusted MoCA trajectory with taVNS; complete variance data were unavailable for a pooled precision estimate | Very low | The effect on longitudinal cognitive screening scores is highly uncertain and should not be equated with a standardized PND diagnosis. |

## GRADE explanatory footnotes

a. Downgraded one level for risk of bias because both contributing trials were rated Some concerns overall. For Zhou and Li, the principal concerns arose from incomplete allocation-concealment reporting, uncertainty around intervention deviations/assignment analysis, and unavailable dated analysis plans; both had Low judgments for missing outcome data and outcome measurement. Neither trial was rated High overall.

b. Downgraded one level for indirectness because the synthesis combined consensus-defined dNCR and investigator-defined traditional POCD, which are clinically related but diagnostically non-equivalent outcome constructs. The pooled estimate was therefore treated as an exploratory cross-construct summary rather than an effect estimate for a single standardized cognitive endpoint.

c. Downgraded one level for imprecision because only 44 events were observed among 220 participants, and the information size remains limited despite a confidence interval excluding the null.

d. Downgraded one level for risk of bias because Shi et al. was rated Some concerns overall, driven by the unavailable dated analysis plan for multiple reported analyses. The randomization, intervention-deviation, missing-data, and outcome-measurement domains were rated Low; administrator awareness alone did not determine the domain judgment.

e. Downgraded one level for imprecision because only 28 POD events occurred and the upper confidence limit approached 1.00.

f. Downgraded one level for risk of bias because Ma et al. was rated Some concerns overall: random-sequence generation was not described, participant/provider masking and trial-context deviations were incompletely reported, and a dated repeated-measures analysis plan was unavailable. Allocation concealment, near-complete outcome availability with a consistent multiple-imputation sensitivity analysis, and blinded outcome assessment were considered Low risk.

g. Downgraded one level for indirectness because a longitudinal MoCA trajectory is not equivalent to a consensus-defined PND diagnosis or a comprehensive neuropsychological assessment.

h. Downgraded one level for imprecision because complete variance estimates and confidence intervals for the adjusted month-1 and month-3 MoCA differences were unavailable; the estimate came from one small trial.

*Abbreviations: CI, confidence interval; dNCR, delayed neurocognitive recovery; MD, mean difference; MoCA, Montreal Cognitive Assessment; PND, perioperative neurocognitive disorders; POD, postoperative delirium; POCD, postoperative cognitive dysfunction; RCT, randomized controlled trial; RR, risk ratio; taVNS, transcutaneous auricular vagus nerve stimulation.*

**Supplementary Data S6. Study-level data used for the exploratory quantitative synthesis**

| **Study** | **Outcome** | **taVNS events** | **taVNS total** | **Control events** | **Control total** |
| --- | --- | --- | --- | --- | --- |
| Zhou et al., 2022 | dNCR at postoperative week 1 | 6 | 60 | 16 | 59 |
| Li et al., 2026 | Investigator-defined early POCD | 5 | 49 | 17 | 52 |

**Supplementary Code S7. Python code used for the exploratory quantitative synthesis**

from math import log, exp, sqrt, erfc

studies = [
 {"study": "Zhou et al., 2022", "a": 6, "n1": 60, "c": 16, "n0": 59},
 {"study": "Li et al., 2026", "a": 5, "n1": 49, "c": 17, "n0": 52},
]

z_975 = 1.959963984540054
\# Two-sided 97.5th percentile of Student t with df=1.
t_975_df1 = 12.7062047361747

\# Study-level log risk ratios and sampling variances.
for s in studies:
 rr = (s["a"] / s["n1"]) / (s["c"] / s["n0"])
 var = 1/s["a"] - 1/s["n1"] + 1/s["c"] - 1/s["n0"]
 s["log_rr"] = log(rr)
 s["var"] = var


def pooled_at_tau2(tau2):
 weights = [1 / (s["var"] + tau2) for s in studies]
 mu = sum(w * s["log_rr"] for w, s in zip(weights, studies)) / sum(weights)
 q = sum(w * (s["log_rr"] - mu)**2 for w, s in zip(weights, studies))
 return mu, weights, q


\# Common-effect inverse-variance estimate.
mu_common, w_common, q_common = pooled_at_tau2(0.0)
se_common = sqrt(1 / sum(w_common))
rr_common = exp(mu_common)
ci_common = (
 exp(mu_common - z_975 * se_common),
 exp(mu_common + z_975 * se_common),
)
z_value = mu_common / se_common
p_value = erfc(abs(z_value) / sqrt(2))
df = len(studies) - 1
i2 = max(0.0, (q_common - df) / q_common) * 100 if q_common > 0 else 0.0

\# Paule-Mandel tau-squared: solve Q(tau-squared) = k - 1.
if q_common <= df:
 tau2_pm = 0.0
else:
 lo, hi = 0.0, 1.0
 while pooled_at_tau2(hi)[2] > df:
 hi *= 2.0
 for _ in range(200):
 mid = (lo + hi) / 2.0
 if pooled_at_tau2(mid)[2] > df:
 lo = mid
 else:
 hi = mid
 tau2_pm = (lo + hi) / 2.0

mu_random, w_random, q_random = pooled_at_tau2(tau2_pm)
se_random = sqrt(1 / sum(w_random))
rr_random = exp(mu_random)
ci_random = (
 exp(mu_random - z_975 * se_random),
 exp(mu_random + z_975 * se_random),
)

\# Standard Hartung-Knapp interval using the Paule-Mandel weights.
hk_scale = q_random / df
se_hk = sqrt(hk_scale / sum(w_random))
ci_hk = (
 exp(mu_random - t_975_df1 * se_hk),
 exp(mu_random + t_975_df1 * se_hk),
)

print({
 "common_effect_RR": rr_common,
 "common_effect_95CI": ci_common,
 "P": p_value,
 "Q": q_common,
 "I2_percent": i2,
 "tau2_Paule_Mandel": tau2_pm,
 "PM_random_effects_RR": rr_random,
 "PM_random_effects_95CI": ci_random,
 "Hartung_Knapp_95CI": ci_hk,
})

# Supplementary Table S8. Study-level adverse-event reporting in included perioperative randomized trials

| **Study** | **Adverse-event definition** | **Ascertainment method** | **taVNS-group events** | **Control-group events** | **Withdrawals due to adverse events** | **Serious adverse events** | **Cardiovascular/autonomic adverse events** | **Reporting limitations** |
| --- | --- | --- | --- | --- | --- | --- | --- | --- |
| Zhou et al., 2022 | Table 3 defines bradycardia (HR<50/min or >30% decrease), hypotension (SBP<90 mmHg or >30% decrease), tachycardia (HR>120/min or >30% increase), hypertension (SBP>180 mmHg or >30% increase), and PONV; general AE framework NR. | Intraoperative physiologic monitoring and Table 3 event reporting; a separate AE ascertainment protocol NR. | Bradycardia 7/62; hypotension 8/62; tachycardia 7/62; hypertension 17/62; PONV 18/62. Authors reported no stimulation-related pain, itching, or headache; counts NR. | Bradycardia 5/62; hypotension 11/62; tachycardia 10/62; hypertension 20/62; PONV 16/62. | NR. Two stimulation sessions were terminated early and one participant received combined IV–inhalation anesthesia; AE attribution/reasons NR. | NR; serious-AE definition/results not specifically reported. | The listed cardiovascular events were perioperative events and were not attributed to stimulation; event definitions are provided in Table 3. | Group-level perioperative event counts were reported, but causal attribution to the device was not. Authors' statement about no pain/itching/headache lacks group-specific counts; unreported serious events are not interpreted as zero. |
| Shi et al., 2026 | Specific AE definition NR. | Participant self-report, medical-record review, and investigator observation. | NR; the article does not provide group-specific stimulation-related AE counts/results. | NR; the article does not provide group-specific stimulation-related AE counts/results. | All 150 completed; AE-specific withdrawal status NR. | NR. | Group-specific cardiovascular/autonomic stimulation-AE counts NR. | The ascertainment channels are stated, but no group-specific AE results are reported; surgical complications are not assumed to be device-related. |
| Li et al., 2026 | Structured stimulation-AE definition NR. | Stimulation-AE ascertainment method NR. | Device-related AE counts NR. PONV was 3/49 as a clinical secondary outcome, not attributed as a device AE. | Device-related AE counts NR. PONV was 12/52 as a clinical secondary outcome, not attributed as a device AE. | Two active-arm withdrawals: procedure canceled and refusal of postoperative stimulation; neither was reported as AE-related. | NR. | Cardiovascular/autonomic device-AE counts NR. | The article does not provide structured AE definitions, systematic ascertainment details, or group-specific device-related AE counts. PONV is not substituted for a stimulation-related AE. |
| Ma et al., 2026 | AEs graded using CTCAE v5.0; postoperative complications classified with Clavien–Dindo. | Exact AE ascertainment procedure NR; safety outcomes were collected as AEs, SAEs, and postoperative complications. | 2/49 (4.1%); events described as mild local auricular erythema/transient tingling overall; type-by-arm counts NR. | 2/50 (4.0%); events described as mild local auricular erythema/transient tingling overall; type-by-arm counts NR. | Four active-arm participants withdrew consent before intervention; not reported as AE-related. | No serious AEs were observed in the analytic cohort. | Cardiovascular/autonomic stimulation-related events NR. | Overall AE counts are explicitly reported; the distribution of local reaction types between groups and detailed ascertainment are NR. No Clavien–Dindo grade ≥II postoperative complications were documented in the analytic cohort. |

NR, not reported in the four primary trial reports. NR does not indicate that no events occurred.
